# Phosphatidylinositol Monophosphates Regulate Optimal Vav1 Signaling Output

**DOI:** 10.3390/cells8121649

**Published:** 2019-12-16

**Authors:** Sonia Rodríguez-Fdez, Carmen Citterio, L. Francisco Lorenzo-Martín, Jesús Baltanás-Copado, Clara Llorente-González, Senena Corbalán-García, Miguel Vicente-Manzanares, Xosé R. Bustelo

**Affiliations:** 1 Centro de Investigación del Cáncer, CSIC–University of Salamanca, 37007 Salamanca, Spain; soniarf@usal.es (S.R.-F.); carmen.citterio@gmail.com (C.C.); Fran_lm@usal.es (L.F.L.-M.); clara.llorente@usal.es (C.L.-G.); miguel.vicente@usal.es (M.V.-M.); 2 Instituto de Biología Molecular y Celular del Cáncer, CSIC–University of Salamanca, 37007 Salamanca, Spain; 3 Centro de Investigación Biomédica en Red de Cáncer (CIBER*ONC*), CSIC–University of Salamanca, 37007 Salamanca, Spain; 4 Department of Biochemistry and Molecular Biology, University of Murcia, 30100 Murcia, Spain; jesus.baltanas@um.es (J.B.-C.); senena@um.es (S.C.-G.); 5 Biomedical Research Institute of Murcia, University of Murcia, 30100 Murcia, Spain

**Keywords:** T lymphocytes, Vav, Rho GEFs, Rac1, JNK, NFAT, T cell receptor, phospholipids, PI5P, PI4P

## Abstract

Phosphatidylinositol–5 phosphate (PI5P) and other mono-phosphoinositides (mono-PIs) play second messenger roles in both physiological and pathological conditions. Despite this, their intracellular targets and mechanisms of action remain poorly characterized. Here, we show that Vav1, a protein that exhibits both Rac1 GDP/GTP exchange and adaptor activities, is positively modulated by PI5P and, possibly, other mono-PIs. Unlike other phospholipid–protein complexes, the affinity and specificity of the Vav1–lipid interaction entail a new structural solution that involves the synergistic action of the Vav1 C1 domain and an adjacent polybasic tail. This new regulatory layer, which is not conserved in the Vav family paralogs, favors the engagement of optimal Vav1 signaling outputs in lymphocytes.

## 1. Introduction

Mono-PIs were initially considered as mere biosynthetic precursors of more complex PIs such as PI(4,5)–bisphosphate (PI(4,5)P_2_). However, it is now becoming apparent that they play signaling roles as second messengers in a number of physiological and pathological processes [1,2,3]. For example, recent results have shown that PI5P conveys compartmentalized signals at the plasma membrane, endocytic membranes, and the nucleus in several cell types [1,2,4,5,6,7,8,9]. The effects of these lipids are relayed via the interaction with intracellular targets bearing specialized PI-binding regions such as the pleckstrin homology (PH), plant homeodomain (PHD), Vps27–Hrs–Stam (VHS), Phox homology (PX), and Fab1–Yotb–Vac1–EEA1 (FYVE) domains [2,10].

**Figure 1:**
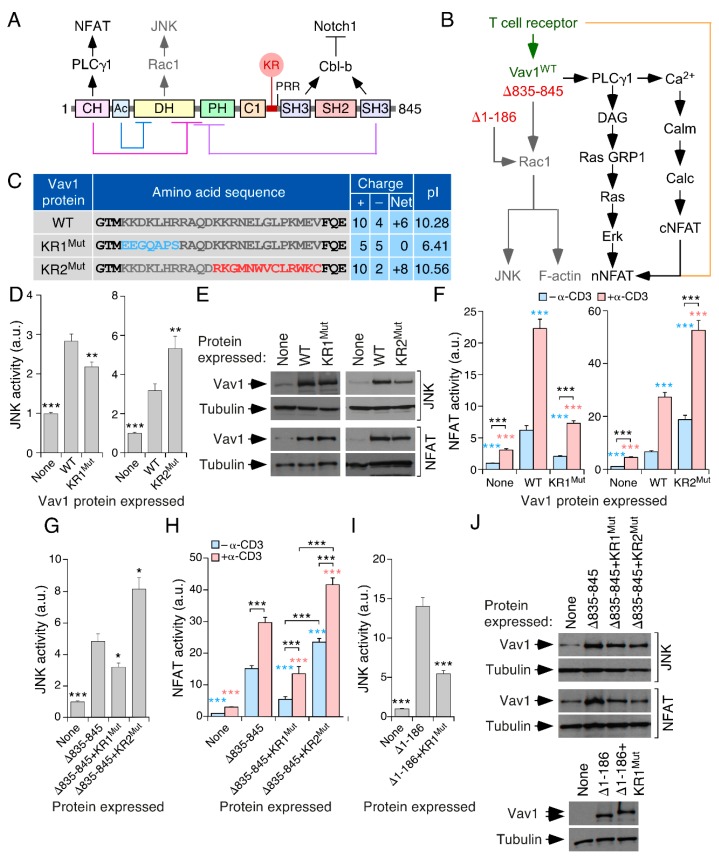
The KR region contributes to Vav1 optimal signaling output in lymphocytes. (**A**) Schematic representation of the Vav1 structure. The main effector pathways of Vav1 in T cells are shown on top. The intramolecular interactions that mediate the inhibition of Vav1 activity in the nonphosphorylated state are depicted at the bottom. (**B**) Schematic representation of the Vav1-dependent pathways in T cells. The Rac1-dependent pathway that is directly stimulated by the GDP/GTP exchange factor (GEF) activity of Vav1 is shown in gray. The Vav1 catalysis-independent pathway involved in the activation of NFAT is shown in black. Synergies established with parallel, T cell receptor (TCR)/CD3–triggered pathways are shown in brown. The WT and constitutively active versions of Vav1 used in this study are shown in green and red, respectively. DAG, diacylglycerol; GRP, GDP releasing factor; Calm, calmodulin; Calc, calcineurin; cNFAT, cytosolic NFAT; nNFAT, nuclear NFAT. (**C**) Amino acid sequence, number of positively (+) and negatively (–) charged residues, and isoelectric point (pI) of the indicated versions of the Vav1 KR region used in these experiments. (**D**) Activation of c-Jun N-terminal kinase (JNK) by indicated Vav1 proteins in nonstimulated and TCR-stimulated Jurkat cells. Data represent the mean ± SEM. Statistical values calculated using the Mann–Whitney U test are given relative to the data obtained with Vav1^WT^. *n* = 6 (left panel, each performed in triplicate) and 4 (right panel, each performed in duplicate) independent experiments. (**E**) Representative immunoblot analysis showing the abundance of the ectopically expressed Vav1 proteins used in panels D (top blot) and F (third blot from top) of this figure. The expression of endogenous tubulin α was used as internal loading control in each case (the type of assay from which these extracts were derived is indicated on the right). (**F**) Activation of NFAT triggered by indicated Vav1 proteins in nonstimulated and CD3-stimulated T cells. Data represent the mean ± SEM. Statistical values were obtained using the Mann–Whitney U test. Blue and salmon asterisks indicate the significance level compared with nonstimulated and TCR-stimulated Vav1^WT^-expressing cells, respectively. Black asterisks refer to the *p*-values obtained between the indicated experimental pairs (in brackets). *n* = 3 independent experiments, each performed in triplicate. (**G**,**H**) Activation of JNK (**G**) and NFAT (**H**) by the indicated Vav1 proteins in Jurkat cells either untreated (**G**,**H**) or stimulated with antibodies to CD3 (**H**). Data represent the mean ± SEM. Statistics were carried out as above relative to the values obtained with Vav1^Δ835–845^-expressing nonstimulated (blue asterisks) and stimulated (salmon asterisks) cells, as well as between the indicated experimental pairs (in brackets, black asterisks). *n* = 3 (**G**, each performed in duplicate) and 4 (**H**, each performed in triplicate) independent experiments. (**I**) Activation of JNK by indicated Vav1 proteins in nonstimulated cells. Data represent the mean ± SEM. Statistical values were obtained using the Mann–Whitney U test and are given relative to the data obtained with Vav1^Δ1–186^-expressing cells. *n* = 3 independent experiments, each performed in triplicate. (**J**) Representative example of the abundance of the indicated Vav1 proteins and tubulin α (loading control) in JNK and NFAT assays performed in panels G (four top blots) to I (two bottom blots).

Vav1 is a hematopoietic-specific protein whose activity is essential for the development, positive selection, negative selection, and effector functions of T lymphocytes [11,12,13,14,15,16]. This signaling protein works both as a Rho GDP/GTP exchange factor (GEF) and as an adaptor-like protein (Figure 1A) [15,16,17,18,19]. The former function catalyzes the rapid transition of Rac1 and related GTPases from the inactive (GDP-bound) to the active (GTP-bound) state during cell signaling (Figure 1A,B). This activity is mediated by the catalytic Vav1 Dbl homology (DH) domain in a concerted manner with the adjacent PH and atypical C1 domains (Figure 1A) [15,16,20,21]. The adaptor functions are more variegated in terms of effector domains and biological outputs involved [15,16]. For example, the Vav1 calponin homology (CH) domain is involved in the engagement of a phospholipase Cγ (PLCγ)-dependent pathway that triggers the stimulation of the nuclear factor of activated T cells (NFAT) [15,16,18,22] (Figure 1A,B). By contrast, the Vav1 SH3 domains mediate a Cbl–b-dependent tumor suppressor mechanism that promotes the degradation of the active signaling fragment of Notch1 in immature T cells [23] (Figure 1A).

The biological activity of Vav1 is tightly controlled by an intramolecular, tyrosine phosphorylation-dependent mechanism. In the nonphosphorylated state, the protein adopts a close conformation owing to the interaction of the Vav1 CH, an acidic (Ac), and most C-terminal SH3 (CSH3) domains with both the DH and PH regions (Figure 1A). These interactions occlude the effector surfaces of Vav1, leading to the inhibition of its signaling output in naïve cells. Upon cell stimulation, the phosphorylation of Vav1 on several tyrosine residues present in the Ac, C1, and CSH3 domains leads to the release of those autoinhibitory interactions, the exposure of the effector sites of the molecule, and full Vav1 activation [15,16,19,24,25].

Given its multidomain structure (Figure 1A), it is possible that other regulatory mechanisms could contribute to regulate the overall Vav1 signaling output. In agreement with this possibility, it has been shown that protein–protein interactions mediated by the Vav1 SH3 domains contribute to the tethering of the molecule to the plasma membrane upon T cell stimulation [16]. In line with previous data with other PH containing proteins [10], it has been long assumed that Vav1 could be also regulated by direct phospholipid binding. Earlier reports indeed indicated that the catalytic activity of the protein could be stimulated by the binding of PI(3,4,5)–triphosphate (PIP_3_) to the Vav1 PH [26]. However, subsequent biochemical and cell-based experiments demonstrated that this is not the case [27,28]. In fact, recent genetic analyses indicate that Vav1 is located upstream of phosphatidylinositol 3-kinase in lymphocytes [16,29,30].

In this study, we report that Vav1 is a target for PI5P and other mono-PIs. This interaction is mediated by a noncanonical mechanism that involves the atypical Vav1 C1 domain and an adjacent lysine-rich (KR) region. We also provide evidence indicating that this new regulatory layer favors optimal signaling output of the protein during lymphocyte signaling.

## 2. Materials and Methods

### 2.1. Mammalian Expression Vectors

All the Vav family constructs used in this work encode versions of the murine species and were DNA sequence-verified in our Genomics Facility. Plasmids expressing Vav1^WT^ (pJLZ52), Vav1^Δ1–186^ (pMJC10), enhanced green fluorescent protein (EGFP)–Vav1^WT^ (pSRM3), EGFP–Vav1^Δ1–186^ (pNM108), EGFP–Vav1^Δ835–845^ (pMB6), and His-tagged Vav2^WT^ (pAO1) were previously described [20,21,24,31,32]. The pNF–AT–Luc and the pSRE–luc plasmid were obtained from Addgene (Watertown, MA, USA), the pFR–Luc and pFA2–cJun plasmids from Stratagene (now, Agilent Technologies, Santa Clara, CA, USA), and the pRL–SV40 plasmid from Promega (Madison, WI, USA). The plasmid encoding the Life-Act was obtained from M.A. del Pozo (Centro Nacional de Investigaciones Cardiovasculares, Madrid, Spain). The plasmids encoding wild-type IpgD (pRK5–Myc–IpgD), the catalytically inactive IpgD mutant (pRK5–Myc–IpgD–C438S), and the PI5P reporter (pEGFP–PHD3x) were all obtained from B. Payrastre (INSERM U1048, Toulouse, France). The vectors encoding the bioreporters for PI3P (pPX–YFP) and PI4P (pFAPP1–GFP) were obtained from J.E. Galán (Yale University, New Haven, USA). The specificity of each of those bioreporters has been described in previous publications [33,34].

The rest of the plasmids encoding Vav1 mutant proteins were generated in this work by site-directed mutagenesis using the high-fidelity NZYProof DNA polymerase (Cat. #14601, NZYTech, Lisbon, Portugal) and the appropriate combination of mutation-bearing oligonucleotides (Table S1). The generation of the plasmids encoding the Vav1 KR1^Mut^ (pSRF10) and KR2^Mut^ (PSRF9) mutants required a two-step site-directed mutagenesis strategy. In the first step, we deleted (in the case of KR1^Mut^) or inserted (in the case of KR1^Mut^) a single nucleotide before the first codon of the nucleotide sequence that we want to mutate. The second mutagenesis step involved the insertion (in the case of KR1^Mut^) and deletion (in the case of KR1^Mut^) of a nucleotide to recover the reading frame at the end of the region that was mutated. In all of these cases, the pJLZ52 plasmid was used as the first DNA template for the PCR reaction. The plasmids encoding Vav1 Δ835–845+KR1^Mut^ (pSRF79) and Vav1 Δ835–845+KR2^Mut^ (pSRF37) were generated as above using the pSRF49 plasmid as a template. The vector encoding Vav1 Δ1–186+KR1^Mut^ (pSRF23) was generated as above using the pMJC10 plasmid as a template. The expression vectors encoding Vav1^Δ835–845^ (pSRF49), Vav1^E591K^ (pSRF41), Vav1^E598K^ (pSRF40), and Vav1^G691V^ (pSRF46) were generated using the oligonucleotides shown in Table S1 and the pJLZ52 plasmid as a template. The vectors encoding Vav1^E591K + E5988K^ (pSRF88) and Vav1^3xEK^ (pSRF97) were generated using the indicated nucleotides (Table S1) and the plasmids pSRF41 and pSRF88, respectively, as templates.

To generate the plasmids encoding EGFP–tagged Vav1 KR^Mut^, the vectors pSRF10 and pSRF9 were digested with KpnI and EcoRI to liberate a fragment of the mouse *Vav1* cDNA encoding the KR region. Upon purification, the cDNA fragment was ligated into the appropriate Vav1-encoding expression vector that was previously linearized with KpnI and EcoRI. Specifically, the KpnI–EcoRI fragments used to generate the EGFP–Vav1 KR1^Mut^–(pSRF20) and EGFP–Vav1 KR2^Mut^–(pSRF17) encoding vectors were liberated from the pSRM3 plasmid, those for the EGFP–Vav1 Δ1–186+KR1^Mut^ (pSRF21) and EGFP–Vav1 Δ1–186+KR2^Mut^ (pSRF18) from pNM108, and those for EGFP–Vav1 Δ835–845+KR1^Mut^ (pSRF22) and EGFP–Vav1 Δ835–845+KR2^Mut^ (pSRF19) from pMB6.

For the generation of the plasmid encoding the His-tagged Vav2+KR^Vav1^ chimera, we used a synthetic double stranded DNA flanked by KpnI and XbaI sites (Cat. 16AB33NC_1918158, Thermo Fisher Scientific, Waltham, MA, USA) that encoded an altered version of Vav2 in which its amino acids 572 to 588 were replaced by the *Vav1* sequence encoding the amino acid sequence between the 564 and the 604 residues. The sequence was then cloned into the KpnI–XbaI–linearized pAO1 plasmid that encoded the full-length Vav2 protein.

The plasmid encoding Vav1^CAAX^ (pSRF93) was obtained in two mutagenic steps. The first one involved an inverse PCR using pJLZ52 as a template and the primers shown in Table S1 to introduce the most C-terminal sequence (CVLS) of the H-Ras CAAX box. In the second step, the nucleotides encoding the sequence of the five H-Ras amino acids located N-terminally to the CVLS box (CMSCK) were inserted via a site-directed insertional mutagenesis approach using the former plasmid as a template and the second pair of primers indicated in Table S1. The plasmids encoding Vav1 KR1^Mut+CAAX^ (pSRF98) and Vav1^G691V+CAAX^ (pSRF96) were obtained by site-directed mutagenesis using the pSRF93 as a template and the indicated primers (Table S1).

### 2.2. Bacterial Expression Vectors

The plasmids encoding maltose binding protein (MBP)–Vav1 DH–PH–C1–KR (pXRB7), MBP–Vav1 PH (pJLZ2), MBP–Vav1 C1–KR (pXRB1), and MBP–Vav3 C1–KRL (pNM128) were described previously [20,21,24]. The bacterial expression plasmid encoding the MBP–Vav1 C1 (pSRF120) was obtained by introducing a stop codon after the residue 570 of Vav1 by site-directed mutagenesis using the primers indicated in Table S1 and the pXRB1 template. To generate the plasmid encoding the MBP–Vav1 KR (SRF126), a *Vav1* cDNA fragment encoding the KR region flanked by BglII and BamHI restriction sites was PCR-amplified from the pJLZ52 vector using the primers indicated in Table S1 and, upon purification, cloned into the BamHI-linearized pMAL-c vector (New England Biolabs, Ipswich, MA, USA). The plasmids encoding MBP–Vav1 C1–KR1^Mut^ (pSRF149) and MBP–Vav1 C1–KR^3xEK^ (pSRF148) were generated using the plasmid pXRB1 as a template and the mutagenesis strategies described above for the generation of the analogous mutations in full-length Vav1. The same strategy was used to generate the vectors encoding MBP–Vav1 KR1^Mut^ (pSRF137) and MBP–Vav1 KR^3xEK^ (pSRF144), although, in this case, we utilized the pSRF126 plasmid as a template.

### 2.3. Immunological Reagents

The homemade polyclonal antibody to the Vav1 DH domain (Ref. 302–5) was raised in rabbits using an MBP–Vav1 DH fusion protein purified from *E. coli* according to standard techniques. The antibodies to the Vav1 KR region (Ref. 97) and the tyrosine phosphorylated version of Vav1 Y^174^ phosphopeptide (Ref. 613) were raised in rabbits using appropriate nonphosphorylated and phosphorylated synthetic peptides, respectively [24,35,36]. Other antibodies used in this study include those recognizing human CD3 (UCHT1 clone, Cat. #217570; Merk–Millipore, Burlington, MA, USA), tubulin α (Cat. #CP06; Calbiochem, San Diego, CA, USA), polyhistidine residues (Cat. #H–1029; Sigma, St. Louis, MO, USA), chicken IgM (Cat. #8300–01; Southern Biotech, Birmingham, AL, USA), MBP (Cat. #M–6295; Sigma), the Myc epitope (Cat. #M5546; Sigma), and GST (Cat. #G1160; Sigma). Rhodamine-labeled phalloidin was obtained from Invitrogen (Cat. #R415; Carlsbad, CA, USA).

### 2.4. Phylogenetic Analyses

Amino acid sequences from Vav family proteins were obtained from the UniProt database (National Center for Biotechnology Information, Bethesda, MD, USA) and aligned using the multiple sequence alignment by the log-expectation algorithm [37,38] in the Jalview software [39]. The PhyML software was used to build the phylogenetic trees based on the maximum-likelihood principle [40].

### 2.5. Calculation of Isoelectric Points

Isoelectric points were calculated using the ExPASy ProtParam (Swiss Institute of Bioinformatics, Lausanne, Switzerland).

### 2.6. Cell Culture and Treatments

Wild-type (WT) Jurkat and Raji cells were obtained from the ATCC (Old Town Manassas, VA, USA). Jurkat *VAV1*^−/−^ were provided by Dr. R.T. Abraham [41]. *VAV1* knockdown Jurkat cells (shRNA TRCN000007750) were described previously [23]. All cell lineages were grown in RPMI-1640 medium supplemented with 10% fetal calf serum, 1% L-glutamine, penicillin (10 μg/mL), and streptomycin (100 μg/mL). DT40 cells were obtained from the Riken Bioresource Center (Kyoto, Japan) and grown in RPMI-1640 medium supplemented with 10% fetal calf serum, 1% chicken serum, 0.1 mM β–mercaptoethanol, 1% L-glutamine, penicillin (10 μg/mL), and streptomycin (100 μg/mL). COS1 cells were grown in DMEM supplemented with 10% fetal calf serum, 1% L-glutamine, penicillin (10 μg/mL), and streptomycin (100 μg/mL). All tissue culture reagents were obtained from Gibco (Waltham, MA, USA). All cell lines were maintained at 37 °C and a 5% CO_2_ humidified atmosphere. When required, Jurkat and DT40 cells were stimulated for the indicated periods of time with antibodies to human CD3 (7.5 μg/mL) and chicken IgM (5 μg/mL), respectively.

### 2.7. Luciferase Reporter, Immunoprecipitation, and Western Blot Analyses

These experiments were done as described elsewhere [24,42]. When indicated, Jurkat cells were treated with antibodies to CD3 for 2 min.

### 2.8. Confocal Microscopy Analyses

Liposome-transfected COS1 cells and Jurkat cells transfected using the Program #5 of the Neon transfection system (Cat. #MPK5000; Invitrogen) were fixed, stained, and mounted as indicated in the work of [24]. For immune synapse studies, Jurkat cells were transfected as above and, after 36 h, washed with RPMI-1640 medium and then gently mixed with an equal number of Raji cells that had been previously incubated in the presence of both 0.5 µg/mL of superantigen E (Cat. #ET404; Toxin Technology, Sarasota, FL, USA) and 10 µM of the 7–amino–4–chloromethylcoumarin membrane permeable cell tracker (Cat. #C2110; Invitrogen) for 1 h at 37 °C in serum free media. After 15 min, cells were plated on 12 mm diameter, poly–D–lysine–coated coverslips, allowed to settle, and further processed for microscopy analyses as indicated above. When indicated, cells were incubated overnight with anti-Myc (1:1500 dilution) after the blocking step, washed thrice, and incubated 30 min with the Alexa 647-labeled donkey anti-mouse IgG antibody (Cat. # A31571, 1:500 dilution; Life Technologies, Carlsbad, CA, USA) before proceeding with the subsequent steps.

For the experiments using live cells, the Jurkat cells were transfected with 30 µg of the appropriate Vav1-encoding expression plasmid plus 15 µg of pmCherry–LifeAct. After 36 h, cells were washed in RPMI-1640 medium, and allowed to adhere onto poly–L–lysine coated coverslips (1:100 dilution incubated overnight). Upon mounting the coverslips onto Attofluor open chambers (Cat. #A7816; Invitrogen), the cells were maintained in 500 µL of RPMI-1640 medium at 37 °C on the microscope stage. Upon addition of equal numbers of superantigen E-loaded Raji cells to the chambers, the formation of Jurkat–Raji conjugates was recorded using a TCS SP5 confocal microscope (Leica, Wetzlar, Germany) and images were processed using the LAS AF software (version 2.6.0.72266, Leica).

### 2.9. Quantification of Confocal Images

Profiles of fluorescence intensity and levels of protein colocalization along the indicated axes of the cell images were obtained using the ImageJ software (National Institutes of Health, Bethesda, MD, USA). The ratio of Vav1 protein at the plasma membrane was calculated creating a mask with the phalloidin staining to discriminate between the fluorescence of Vav1 at the membrane (coincident with phalloidin-positive structures) and the cytoplasmic fraction (noncoincident with phalloidin-positive F-actin). The orthogonal reconstruction of the contact area between Jurkat–Raji conjugates was carried out using the 3D module of the LAS X software (Leica). The polymerization of F-actin was determined using the ImageJ plug-in previously described [43]. In brief, the rhodamine–phalloidin fluorescence present in either the contact area or the adjacent area (in the case of peripheral ruffling) of the immune synapse was compared with nonengaged membrane areas of both the T cell and the superantigen-presenting cell membrane. The same approach was used to calculate Vav1 polarization to the immune synapse, comparing the fluorescence within the contact area to the signal in other areas of the T cell.

### 2.10. Subcellular Fractionation

20 × 10^6^ exponentially growing Jurkat cells were electroporated as for luciferase reporter assays. After 24 h, cells were either left nonstimulated or stimulated with anti-CD3 for 2 min. Cells were then centrifuged; resuspended in 1 mL of hypotonic buffer [10 mM Tris–HCl (pH 7.5), 38 mM sodium chloride, 1 mM sodium orthovanadate (Cat. #S6508; Sigma), 1 mM sodium fluoride (Cat. #S7920; Sigma), and CØmplete Protease Inhibitor Cocktail (Cat. #11836145001; Roche, Penzberg, Germany)]; and kept on ice for 5 min. After five freeze/thaw cycles (4 min at −80 °C and 20 s at 42 °C), cells were centrifuged at 14,000 rpm for 10 min at 4 °C and the pellets were discarded. The supernatants were then centrifuged in an ultracentrifuge (Optima TL Ultracentrifuge; Beckman Coulter, Brea, CA, USA) at 60,000 rpm for 1 h at 4 °C using polycarbonate centrifuge tubes (Cat. #349622; Beckman Coulter). The supernatant was considered as the cytoplasmic compartment and Triton X-100 (Cat. #X100; Sigma) was added to a final 1% concentration. The pellet was resuspended again in hypotonic buffer and centrifuged as above. This new pellet was resuspended in hypotonic buffer with 1% Triton X-100 to obtain the plasma membrane fraction. SDS-PAGE buffer was added to all the samples, boiled for 5 min, and analyzed by immunoblot as indicated above.

### 2.11. Purification of Maltose Binding Fusion Proteins

Exponentially growing cultures of *E. coli* DH5α cells were treated with isopropyl β–D–1–thiogalactopyranoside (Cat. # I5502; Sigma) at 25 °C for 90 min, centrifuged at 6000 rpm for 10 min at 4 °C, and resuspended in lysis buffer (10 mM sodium phosphate, 0.25% (*w*/*w*) Tween 20, 30 mM NaCl, 10 mM β-mercaptoethanol, 10 mM EDTA, 10 mM EGTA). The samples were frozen overnight, thawed in ice, disrupted by sonication on ice, centrifuged again, and the supernatant containing the maltose binding fusion proteins subjected to purification using an amylose resin (Cat. #E8021; New England Biolabs). The maltose binding fusion proteins were eluted with 5 mL of 10 mM maltose in column buffer (20 mM Tris–HCl, 200 mM NaCl, 1 mM EDTA, pH 7.4) and 500 µL fractions were serially collected. Proteins were conserved at −20 °C until further use. Protein concentration was quantified on polyacrylamide gels using known concentrations of bovine serum albumin as standard.

### 2.12. Phospholipid Binding Assays

Membranes with spotted phospholipids (PIP strips, Cat. #P–6001; PIP arrays, Cat. #P6100; Echelon Biosciencies, Salt Lake City, UT, USA) were blocked for 1 h in blocking buffer (1% nonfat-dry milk in phosphate-buffered saline solution) at room temperature, incubated overnight with either 1 µg/mL (in the case of PIP strips) or 0.5 µg/mL (in the case of PIP arrays) of indicated maltose binding fusion proteins in blocking buffer at 4 °C, washed three times in PBS-T (phosphate-buffered saline containing 0.1% Tween-20), incubated with an antibody to MBP (1:3000 dilution) in PBS-T for one hour at 4 °C, washed three times in PBS-T, and incubated with the secondary antibody for one hour in PBS-T. After three washes with PBS-T, the protein bound to the lipids was detected by chemoluminiscence (Pierce ECL, Cat. #32106; Thermo Fisher Scientific, Waltham, MA, USA). The dot blots were scanned in ImageJ and the results were fitted into a five-parameter nonlinear regression model using GraphPad Prism (version 6.0; GraphPad Software, San Diego, CA, USA).

### 2.13. Lipid Sedimentation Assays

For the preparation of multilamellar vesicles, chloroform–methanol solutions composed of 1-palmitoyl–2-oleoyl–glycero–3-phosphocholine (POPC, Cat. #850457; Avanti polar lipids, Alabaster, AL, USA); 1-palmitoyl–2-oleoyl–sn–glycero–3-phospho–L–serine (POPS, Cat. #840034; Avanti polar lipids); and either L–α–phosphatidylinositol–4,5-bisphosphate (PI(4,5)P_2_, Cat. #840046; Avanti polar lipids), phosphatidylinositol 3-phosphate (PI3P, Cat. #P–3016; Echelon Biosciences), or phosphatidylinositol 5-phosphate (PI5P, Cat. #P–5016; Echelon Biosciences) in a 75:20:5 proportion, respectively, were mixed and dried under a stream of liquid nitrogen gas. After three extra hours of vacuum drying, the dried lipids were suspended in buffer (25 mM Tris (pH 8.0), 25mM NaCl) and allowed to hydrate above the transition temperature of the lipid mixture for 30–60 min. The lipids were then subjected to three freeze–thaw cycles to obtain a homogeneous mix of multilamellar vesicles. For the lipid sedimentation assays, the proteins (5 µM) were mixed and incubated for 10 min at room temperature in the presence of the lipid mix (1 mM) and glycerol (5%). Afterwards, the mix was centrifuged at 15,300 rpm for 35 min. The supernatants or soluble fraction (S) were separated from the pellet (P) (lipid-bound fraction) and analyzed in 15% SDS/PAGE gels stained with Coomassie Brilliant Blue G–250. Quantification was performed using ImageJ (National Institutes of Health, Bethesda, MD, USA).

### 2.14. Purification of Vav1 from Insect Cells and Lipid Binding Assays

Baculovirus were generated from pFastBac-derivatives (Gibco–BRL; Waltham, MA, USA) encoding full length Vav1 protein [17] and used to infect insect *Sf*9 cells, as indicated elsewhere [20,44]. Protein purification was performed using an affinity chromatography step as indicated before [17,20,44]. Purified proteins (1 µg/mL) were used in PIP strips as indicated above.

### 2.15. Alignment of Amino Acid Sequences

The appropriate amino acid sequences of the indicated proteins were obtained from the UniProt (National Center for Biotechnology Information, Bethesda, MD, USA) or the Simple Modular Architecture Research Tool (SMART) database [45] and aligned using Jalview.

### 2.16. Statistical Analyses

All the statistical analyses were carried out using GraphPad Prism software (version 6.0). The number of replicates and the statistical tests used in each case are indicated in the figure legends. In all cases, the *p*-values were depicted using the * (when < 0.05), ** (when < 0.01), and *** (when < 0.001) notation.

## 3. Results

### 3.1. A Lysine-Rich Region Contributes to Vav1 Optimal Signaling Output in Lymphocytes

Vav1 is the only family member that displays a KR region located between the C1 and the proline rich region (PPR)–NSH3 cassette (Figure 1A and Figure S1). Vav3 also harbors an analogous region, although, in this case, the positively charged amino acids are distributed in a more disperse manner than in Vav1 (Figure S1). The KR region represents a late evolutionary acquisition in the Vav family, as it first becomes apparent in teleosts (Figure S1) [15], the first group of species exhibiting a fully developed adaptive immune system [46]. We generated two frameshift mutations that alter the amino acid sequence of the N- (KR1) and C-terminal (KR2) parts of that region to assess its potential involvement in Vav1 signaling output (Figure 1C). In the case of the KR1, the mutation (KR1^Mut^) leads to a 50% reduction in the number of positively charged amino acids, while it does not alter the total number of negatively charged ones. This causes a change from a basic (10.28) to a more acidic (6.41) isoelectric point (pI) of the entire region (Figure 1C). The frameshift mutation within the KR2 region (KR2^Mut^) leaves the positively charged amino acids intact, while it eliminates 50% of the negatively charged residues present in that region. These changes result in an increase in the pI of the Vav1 KR region from the 10.28 WT value to 10.56 (Figure 1C). Given the implication of Vav1 in T cell development and signaling [16], we next tested the biological activity of the mutant proteins using luciferase reporter-based c-Jun N-terminal kinase (JNK) and NFAT activity assays in the T cell acute lymphoblastic leukemia Jurkat cell line. Vav1 activates JNK in a Rac1-dependent manner [21,24,42] and, therefore, the activity of this serine/threonine kinase is traditionally used as a cell-based readout for the activation level of the Vav1 catalysis-dependent pathways (Figure 1B). By contrast, the stimulation of NFAT by Vav1 is catalysis-independent, requiring the activation of a Vav1 CH domain-dependent effector pathway that includes the engagement of PLCγ and the production of Ca^2+^ and diacylglycerol (Figure 1B) [22,47,48]. It also synergizes with other signaling pathways triggered by the T cell receptor (TCR) in order to elicit optimal NFAT activation levels [22,47,48] (Figure 1B). As comparative controls, we used Vav1^WT^ and versions of the protein with C- (Vav1^Δ835–845^) and N-terminal deletions (Vav1^Δ1–186^). Because of the loss of the inhibitory CSH3, Vav1^Δ835–845^ can stimulate both JNK and NFAT activities in a phosphorylation-independent and constitutive manner [24] (Figure 1B). The hyperactive Vav1^Δ1–186^ is also phosphorylation-independent [21]. However, unlike Vav1^Δ835–845^ and Vav1^WT^, it cannot stimulate the NFAT pathway owing to the absence of the CH domain [21,24,42] (Figure 1B). Given that the activity of these two mutant proteins is phosphorylation-independent [21,24], their inclusion in these experiments allowed us to discriminate whether any alteration in Vav1 signaling induced by the KR region mutations could be related to either the early phosphorylation-dependent activation step mediated by the upstream kinases or to later effector signaling phases of phosphorylated Vav1 (e.g., membrane off-rates, dephosphorylation kinetics). Consistent with a potential regulatory role of the KR region, we observed that the full-length Vav1 KR1^Mut^ and KR2^Mut^ mutant proteins display reduced and enhanced activities when tested in both JNK (Figure 1D,E) and NFAT (Figure 1E,F) assays, respectively. This deregulated activity is observed both in untreated cells and in cells stimulated with antibodies to the CD3 subunit of the TCR (Figure 1D–F). The impact of the mutations in Vav1 activity is much larger in the case of NFAT than in JNK assays, according to fold-change criteria (compare Figure 1D,F). These two frameshift mutants elicit the same effects when incorporated in the context of the Vav1^Δ835–845^ (Figure 1G–H) and Vav1^Δ1–186^ (Figure 1I), indicating that the changes in activity induced by those mutations are not owing to alterations in the initial phosphorylation-dependent activation step of the protein. Immunoblot analyses confirmed that the alterations in the activity found in these experiments are not the result of spurious variations in the abundance of the ectopically expressed proteins (Figure 1J). 

To check whether the effect induced by the KR mutations is cell-type specific, we tested the activity of the Vav1 mutants in both hematopoietic (chicken DT40 B lymphocytes) and nonhematopoietic (monkey COS1 cells) cell lines. In the former case, we found that Vav1 KR1^Mut^ shows WT-like activity when tested in NFAT assays. By contrast, the Vav1 KR2^Mut^ displays an exacerbated activity when tested both in nonstimulated and B cell receptor stimulated DT40 cells (Figure S2A,B). In the case of COS1 cells, we measured the activity of the ectopically expressed Vav1 proteins using as readouts the stimulation of serum responsive factor (SRF) and the induction of membrane ruffling in the transfected cells. These two activities require the GEF activity of Vav1 [21,24]. We observed that the full-length Vav1 KR1^Mut^ and KR2^Mut^ proteins display WT-like activities in those two cell-based readouts (Figure S2C–E). The incorporation of the KR1^Mut^ and KR2^Mut^ mutations does not alter the biological activity of both Vav1^Δ835–845^ (Figure S2E–G) and Vav1^Δ1–186^ (Figure S2E,H,I) in those two assays. These results indicate that the contribution of the KR region to Vav1 activity is probably limited to lymphocytes. Given that Vav2 lacks the KR region (Figure S1) and is uncapable of stimulating the NFAT pathway [49], we finally assessed whether the insertion of the Vav1 KR region in the Vav2 structure could promote the stimulation of this transcriptional factor. This was not the case (Figure S2J,K). Taken together, these results indicate that the role of the KR region is related to the modulation of Vav1 signaling in lymphocytes.

### 3.2. The Effect of the KR Region on Vav1 Activity Is Related to Its Electrostatic Properties

**Figure 2:**
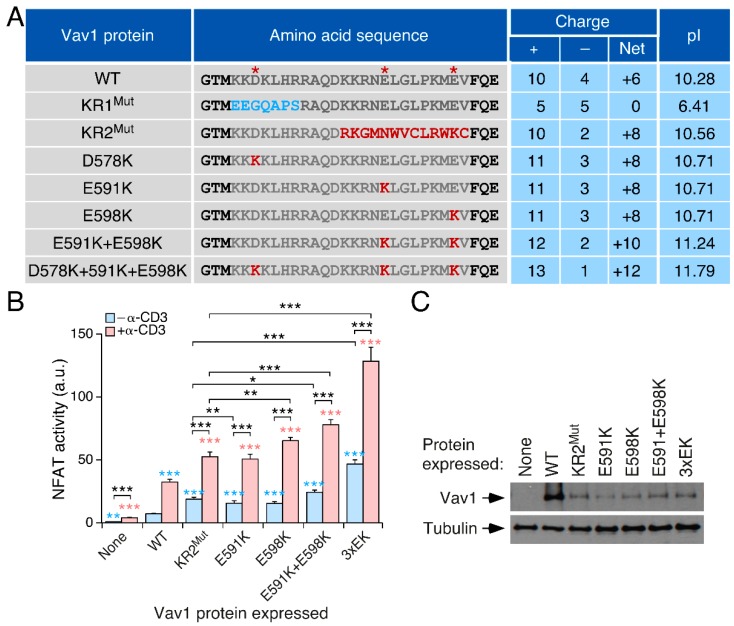
The effect of the KR region on Vav1 activity is related to its electrostatic properties. (**A**) Summary of the Vav1 mutations used and their effect in the overall charge and pI of the KR region. The red asterisks label the negatively charged amino acids that were mutated in these experiments. (**B**) Activation of NFAT by indicated Vav1 mutant proteins and stimulation conditions. Data represent the mean ± SEM. Statistical values obtained using the Mann–Whitney U test are given relative to the data obtained, with *p*-values calculated using the Mann–Whitney U test. Blue and salmon asterisks indicate the *p*-value obtained for each condition relative to that found in both Vav1^WT^-expressing nonstimulated and stimulated cells, respectively. Black asterisks mark the *p*-values obtained between other experimental pairs (shown in brackets). *n* = 3 independent experiments, each performed in triplicate. (**C**) Representative immunoblot showing the abundance of the ectopic proteins in the experiments shown in B.

The impact of the frameshift mutations on the pI of the KR region (Figure 1B), coupled to the fact that polybasic domains such as those present in K-Ras and Rac1 can establish electrostatic interactions with specific phospholipids [50,51], led us to investigate whether the effect of those mutations could be associated with their impact on the overall electrostatic potential of the Vav1 KR region. To this end, we generated single (E^591^, E^598^), double (E^591^ + E^598^), and triple (D^578^ + E^591^ + E^598^) missense mutations in which the indicated negatively charged amino acids of the Vav1 KR region (Figure 2A, red asterisks) were replaced by lysine residues. The impact of each of those mutations in the overall pI of the Vav1 KR region is shown in Figure 2A. We observed that the single E591K mutation (KR pI 10.71) fully recapitulates the effect of the KR2^Mut^ (KR pI 10.56) in terms of upregulation of NFAT activity, both in nonstimulated and TCR-stimulated Jurkat cells (Figure 2B,C). The E598K (pI 10.71) and, to a larger extent, the E591K + E598K mutation (KR pI 11.24), prompt a further elevation of the activity of full-length Vav1 when compared with both the Vav1^E591K^ and the Vav1 KR2^Mut^ mutant versions (Figure 2B,C). However, the most dramatic effect is observed with the full-length Vav1 protein bearing the triple D578K + E591K + E598K mutation (KR pI 11.79) under both nonstimulated and TCR-stimulated conditions (Figure 2B,C). This is specific for the triple mutation, as the single D578K mutation shows WT-like activity when tested in the same assays (SR-F and XRB, data not shown). These results, together with those shown in Figure 1, indicate that there is a direct correlation between the biological activity of Vav1 and the overall pI of its KR region.

### 3.3. The KR Region Is Important for the Localization of Vav1 in Lymphocytes

**Figure 3:**
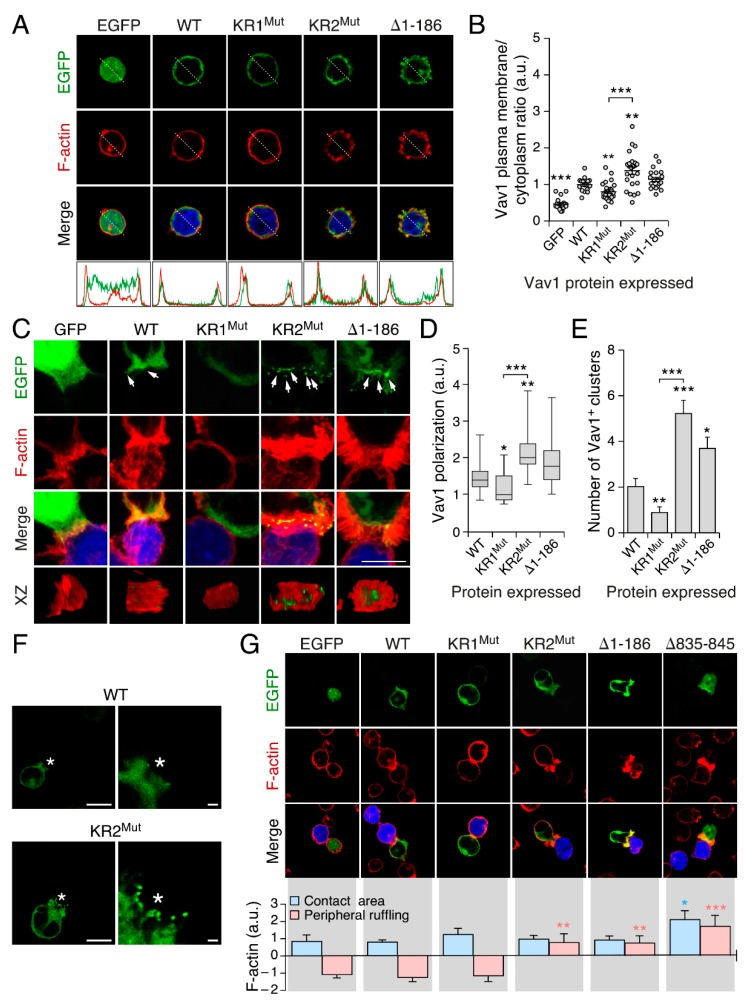
The KR region is important for the localization of Vav1 in lymphocytes. (**A**) *Top*, example of the subcellular localization of enhanced green fluorescent protein (EGFP) (left top panel), indicated EGFP-tagged Vav1 versions (rest of top panels), and F-actin (middle row of panels) in nonstimulated Jurkat cells. Areas of colocalization between EGFPs and F-actin are shown in yellow (bottom panels). Scale bar, 5 µm. *Bottom*, distribution of EGFP (green) and F-actin (red) along the indicated longitudinal axes shown in the immunofluorescence images (white dashed lanes). (**B**) Quantification of the distribution of the indicated Vav1 proteins in juxtamembrane (F-actin positive) and cytosolic (F-actin negative) areas of nonstimulated Jurkat cells from the experiments shown in A. Points represent measurements of individual cells. The mean ± SEM obtained in each condition is also indicated. Statistical values were calculated using Student’s *t* test relative to the data obtained in Vav1^WT^-expressing cells. *n* = 3 independent experiments. (**C**) Distribution of EGFP (top panel on the left), indicated EGFP–Vav1 proteins (top) and F-actin (second row of panels from top) observed in Z–stacked images (three top rows of panels) and orthogonal sections (XZ, bottom panels) of the immune synapse formed by Jurkat cells with superantigen E-loaded Raji cells. EGFP, F-actin, and areas of colocalization between Vav1 proteins and F-actin are shown in green, red, and yellow, respectively. Arrows indicate clusters enriched in Vav1. Scale bar, 10 µm. (**D**) Level of accumulation of indicated EGFP-tagged Vav1 proteins in the immune synapses formed in the experiments shown in C. Boxes, lines inside boxes, and bars represent the 25th to 75th percentiles, the median, and the minimum and maximum values, respectively. Statistical values were calculated using the Mann–Whitney U test relative to the data obtained in Vav1^WT^-expressing cells. *n* = 3 independent experiments. (**E**) Quantification of the number of Vav1 positive clusters found in immune synapses in the experiments shown in C. Data represent the mean ± SEM. Statistical values were obtained using Student’s *t* test relative to the data obtained in Vav1^WT^-expressing cells. *n* = 4 independent experiments. (**F**) Representative example of the localization of indicated EGFP-tagged Vav1 proteins in immune synapses that was captured using time-lapse confocal microscopy. Asterisks indicate the position of the Raji cell that forms the conjugate with the Jurkat cell (green). Scale bar, 10 µm (left panels) and 1 µm (right panels). (**G**) Example (top panels) and quantification (bottom) of the effect of EGFP and indicated EGFP-tagged Vav1 versions (top) in the polymerization of actin both inside and outside the contact area of the immune synapse. Values were obtained comparing the F-actin signal in these areas to the signal in nonengaged regions of both the T and B cell, as detailed in the methods. Histograms represent the mean ± SEM. Statistics were performed using two-way analysis of variance (ANOVA) and Dunnett’s multiple comparison tests using, as reference control, the detection of F-actin inside (blue) and outside (salmon) the contact area in Jurkat cells expressing EGFP–Vav1^WT^. *n =* 3 independent experiments.

The results obtained in the previous section suggested to us that the KR region had to be related to the turnover rates of Vav1 at the plasma membrane. To test this hypothesis, we ectopically expressed the indicated enhanced green fluorescent protein (EGFP)-tagged versions of Vav1 in Jurkat cells and, upon fixation and staining of the F-actin cytoskeleton with rhodamine-labelled phalloidin, we monitored the subcellular localization of each protein under study using confocal microscopy. We found that the EGFP–Vav1 KR1^Mut^ protein shows ratios of colocalization with juxtamembrane F-actin filaments lower than those exhibited by both Vav1^WT^ and Vav1^Δ1–186^ (Figure 3A,B). By contrast, the EGFP–Vav1 KR2^Mut^ protein displays ratios of colocalization with F-actin higher than the WT version (Figure 3A,B). We also observed that the ectopic expression of EGFP–Vav1 KR2^Mut^, but not of EGFP–Vav1^WT^ or EGFP–Vav1 KR1^Mut^, promotes membrane ruffling in the transfected cells (Figure 3A). As previously described [24], this ruffling activity is also detected in the case of EGFP–Vav1^Δ1–186^-expressing Jurkat cells (Figure 3A). Using standard subcellular fractionation analyses, we observed that the EGFP–Vav1 KR2^Mut^ and KR1^Mut^ versions show better and worse binding, respectively, to membrane fractions than Vav1^WT^ in Jurkat cells (Figure S3A,B). Despite this, we could not find any statistically significant difference in the overall phosphorylation level of the interrogated Vav1 proteins in both nonstimulated and TCR-stimulated Jurkat cells (Figure S3C,D). This further suggests that the KR region influences the stability of already activated Vav1 proteins at the plasma membrane.

We performed further microscopy analyses to monitor the influence of the KR region on the localization of Vav1 during the formation of the immune synapse. To this end, EGFP–Vav1-transfected cells were exposed to superantigen-loaded Raji B cells to favor the generation of immune synapses in the T-cell/B-cell interface and, upon fixation and rhodamine–phalloidin staining, the localization of Vav1 and the overall structure of the synapse itself was determined by confocal microscopy. In this assay, we found that EGFP–Vav1^WT^ displays a dispersed, dot-like pattern distribution within the synapse, which is well observed in orthogonal reconstructions of this structure (Figure 3C, second bottom panel from left). Under the same experimental conditions, the full-length EGFP–Vav1 KR1^Mut^ and KR2^Mut^ proteins show reduced and increased localization, respectively, at the immune synapse (Figure 3C–E). The enhanced localization of the KR2^Mut^ protein goes along with an increase in its detection of EGFP^+^ dot-like clusters present right in the center of the synapse (Figure 3C,E). The Vav1 KR2^Mut^ also localizes in dot-like clusters present at the tips of the ruffles that form the peripheral edges of the synapse, a location hardly seen in Vav1^WT^ (Figure 3F). Further analyses of the Jurkat–Raji conjugates revealed that the full-length Vav1 KR2^Mut^ promotes extensive ruffling activity in the peripheral area of the synapse (Figure 3G). This effect, which is not observed in the case of Vav1^WT^ and Vav1 KR1^Mut^ (Figure 3G), is similar to that elicited by both Vav1^Δ1–186^ and Vav1^Δ835–845^ when ectopically expressed in Jurkat cells (Figure 3G). The inclusion of the KR1^Mut^ also promotes a statistically significant reduction in the peripheral ruffling activity induced by these two hyperactive versions of Vav1 in Jurkat cells (Figure S3E). However, the incorporation of the KR2^Mut^ does not induce a significant effect in levels of ruffling activity induced by those two proteins (Figure S3E). Taken together, these results indicate that the KR is important for the localization of Vav1 in F-actin-rich juxtamembrane areas and the immune synapse in the case of nonconjugated and B cell-conjugated Jurkat cells, respectively. They are also consistent with the association of the KR2^Mut^ with the increased Vav1 activity observed in those two experimental conditions.

### 3.4. The Inclusion of the H-Ras CAAX Box Rescues the Activity of Vav1 KR1^Mut^

**Figure 4:**
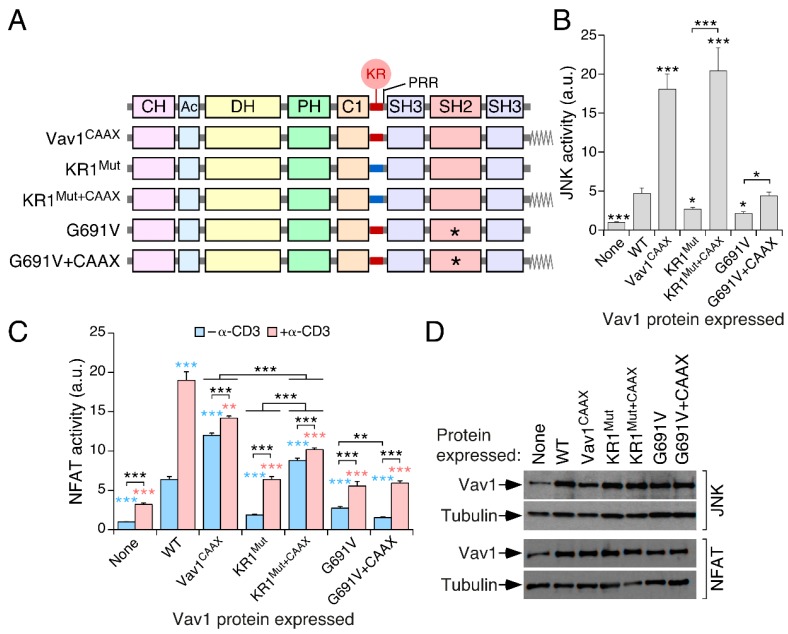
The inclusion of the H-Ras CAAX box rescues the activity of Vav1 KR1^Mut^. (**A**) Depiction of the Vav1 proteins used in these experiments. (**B**) Activation of JNK by indicated Vav1 proteins in nonstimulated Jurkat cells. Data represent the mean ± SEM. Statistical values were obtained using the Mann–Whitney U test and are given relative to the data obtained in Vav1^WT^-expressing cells or the indicated experimental pairs (in brackets). *n* = 3 independent experiments, each performed in triplicate. (**C**) Activation of NFAT by indicated Vav1 proteins in cells that were either nonstimulated or stimulated with antibodies to CD3. Data represent the mean ± SEM. Statistical values were obtained using the Mann–Whitney U test. Blue and salmon asterisks indicate the significance level compared with nonstimulated and TCR-stimulated Vav1^WT^-expressing cells, respectively. Black asterisks refer to the *p*-values obtained between the indicated experimental pairs (in brackets). *n* = 4 independent experiments, each performed in triplicate. (**D**) Representative immunoblots showing the abundance of the ectopic proteins and endogenous tubulin α in the experiments shown in B (two top blots) and C (two bottom blots), respectively.

We next investigated whether the introduction of the C-terminal CMSCKCVLS peptide of H-Ras at the Vav1 C-terminus (Figure 4A) could counteract the effect of the KR1^Mut^. This peptide, which contains the CAAX box that undergoes prenylation and the subsequent endoproteolytic and methylation steps during the normal biosynthetic cycle of the GTPase, promotes the constitutive localization of the experimentally-tagged proteins at the plasma membrane [21,52]. As control, we used the CAAX box-containing versions of Vav1^WT^ and Vav1^G691V^. The latter protein is biologically inactive owing to the presence of a point mutation in the SH2 that eliminates the interaction with upstream kinases and other tyrosine-phosphorylated proteins [21] (Figure 4A). When tested in JNK assays, we observed that the attachment of the CAAX box promotes similar levels of hyperactivation in the case of the WT and KR1^Mut^ versions of Vav1 (Figure 4B). However, such a rescue is not observed when the CAAX box is attached to the Vav1^G691V^ mutant (Figure 4B). This indicates that, despite the membrane tethering induced by the H-Ras CAAX box, Vav1 still needs the SH2-dependent interaction with upstream kinases to become fully activated in T cells.

The results obtained with this collection of CAAX-tagged proteins in the NFAT assays were more complex. In the case of nonstimulated cells, the inclusion of the CAAX box does rescue the activity of the Vav1 KR1^Mut^ version to levels slightly higher to those found in the case of the WT counterpart (Figure 4C). In addition, it elicits higher levels of activity when attached to Vav1^WT^ (Figure 4C). In the case of Jurkat cells stimulated with antibodies to CD3, the CAAX box-containing Vav1^WT^ and KR1^Mut^ proteins only undergo a minor upregulation of the activity already achieved in the nonstimulated conditions (Figure 4C). As a result, the overall activity of these proteins is always lower than that displayed by Vav1^WT^ in stimulated cells (Figure 4C). The CAAX-tagged version of Vav1^G691V^ also remains inactive in the case of TCR-stimulated cells (Figure 4C). The proper expression of the proteins under analysis was corroborated using immunoblots (Figure 4D). These results indicate that the H-Ras CAAX box rescues the biological activity of the KR1^Mut^ version of full-length Vav1.

### 3.5. The Vav1 KR Region Contributes to the Binding of Specific Mono-PIs

**Figure 5:**
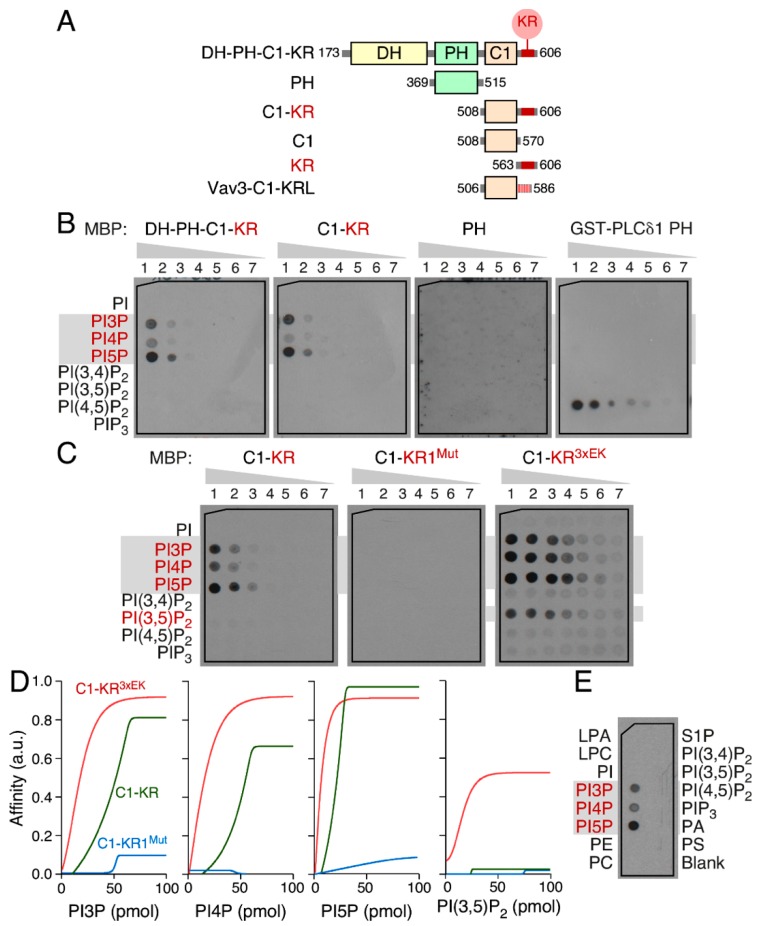
The Vav1 KR region mediates phosphatidylinositol monophosphate binding. (**A**) Depiction of the maltose binding proteins utilized in these experiments. KRL, KR-like. (**B**,**C**) Example of the binding of the indicated MBP (three left panels) and GST (right panel) fusion proteins to phospho-inositides (PIs) (left). The amount of the indicated lipids (left) spotted on the filters was 100 (column 1), 50 (column 2), 25 (column 3), 12.5 (column 4), 6.25 (column 5), 3.13 (column 6), and 1.56 (column 7) pmol. The lipids showing positive binding to the MBP–Vav1 proteins are shaded in gray and colored in red. PI(3,4)P_2_, phosphatidylinositol (3,4)-bisphosphate; PI(3,5)P_2_, phosphatidylinositol (3,5)-bisphosphate; PI(4,5)P_2_, phosphatidylinositol (4,5)-bisphosphate. Similar results were obtained in two (MBP binding assays) and one (GST binding assays) independent experiments. (**D**) Densitometric analysis showing the relative affinity of the indicated MBP–Vav1 proteins with the PIs shown at the bottom. (**E**) Example of the binding of the His-tagged Vav1^WT^ purified from baculovirus-infected *Sf*9 cells to indicated lipids.

The connection between the signaling output of Vav1 and the electrostatic potential of its KR region suggested that this amino acid sequence was probably involved in a phospholipid-dependent anchoring step. To verify this hypothesis, we purified from *E. coli* maltose binding proteins (MBPs) fused to different combinations of the central domains of Vav1 in *E. coli* (Figure 5A and Figure S4A) and tested their affinity to a large variety of phospholipid molecules using protein–lipid blot assays. We found that the MBP–Vav1 DH–PH–C1–KR (Figure 5B, left panel) and the MBP–Vav1 C1–KR (Figure 5B, second panel from left), but not the MBP fused to the Vav1 PH domain (Figure 5B, third panel from left), could bind with progressively weaker affinities to PI5P, PI3P, and PI4P. By contrast, none of the fusion proteins can interact with PI, PI bisphosphate species, and PIP_3_ (Figure 5B). As positive control, the blotting of the same filters with a GST–PLC– δ1 PH domain fusion protein results in the expected binding to PI(4,5)P_2_ (Figure 5B, right panel) [10]. In agreement with our previous cell-based experiments, we observed that the incorporation of the KR1^Mut^ abrogates the binding of the purified MBP–Vav1 C1–KR protein (Figure S4B) to the three mono-PIs (Figure 5C,D). By contrast, the purified MBP–Vav1 C1–KR^3xEK^ (Figure S4B) displays higher binding to those lipids (Figure 5C,D) and, in addition, can recognize PI(3,5)P_2_ (Figure 5C, right panel). The specific affinity of the Vav1 C1–KR domains for PI5P and PI3P was confirmed using independent liposome-binding assays (Figure S4C,D). These results indicate that the Vav1 KR region is important for the interaction with mono-PIs.

To further assess the relevance of this interaction, we investigated the lipid binding activity of a full-length Vav1 protein purified from baculovirus-infected *Sf*9 cells (Figure S4E). As shown in Figure 5E, this protein can bind to PI5P and, to a lesser extent, to PI3P and PI4P. By contrast, the full-length Vav1 protein cannot bind to the rest of lipid compounds included in the blot assays (Figure 5E). Given that nonphosphorylated Vav1 is in an inhibited conformation [17,19,21], these results indicate that the KR region must be surface-exposed even when the protein is in the “closed”, inactive conformation. Consistent with this, we could immunoprecipitate full-length Vav1^WT^ using an antibody to the Vav1 KR region as efficiently as when using antibodies to the catalytic domain in nonstimulated Jurkat cells (Figure S4F).

### 3.6. Phospholipid Binding Requires Cooperation of the Vav1 C1 and KR Regions

**Figure 6:**
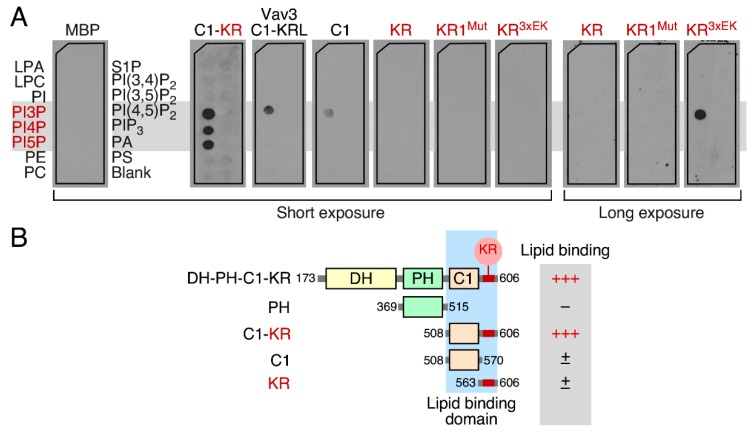
Phospholipid binding requires cooperation of the Vav1 C1 and KR regions. (**A**) Binding of the indicated MBPs (top) to different lipid molecules (shown on the left panel). The lipids that bind to the MBP–Vav1 C1–KR protein are shaded in gray and colored in red. In the case of the KR region, KR1^Mut^, and KR^3xEK^ proteins, we show both short and long exposures of the chemoluminiscence signal. Similar results were obtained in a second independent experiment. LPA, lysophosphatidic acid; LPC, lysophosphocholine; PE, phosphatidyl–ethanolamine; PC, phosphatidylcholine; S1P: sphingosine–1–phosphate; PA, phosphatidic acid; PS, phosphatidylserine. Blank, no lipid spotted. The abbreviations for the rest of the lipids used are described in the legend to Figure 5B. (**B**) Diagram summarizing the results obtained. The lipid binding site of Vav1 is shown as a shaded blue area.

We utilized the filter binding approach described above to get further insights into the organization of this new mono-PI binding region of Vav1. To this end, we purified from *E. coli* new MBP fusions containing either the Vav1 C1 or the KR region alone (Figure 5A and Figure S4G). Interestingly, we observed that the purified MBP–Vav1 C1 (Figure 6A, fourth panel from left) and, to a larger extent, the MBP–Vav1 KR (Figure 6A, fifth panel from left) cannot recapitulate the binding of the MBP–C1–KR (Figure 6A, second panel from left). This is even the case when using the MBP–Vav1 KR^3xEK^ version (Figure 6A, seventh panel from left), although longer exposures of the filters indicate that the latter protein can interact with low affinity with PI3P (Figure 6A, right panel). These experiments also indicate that the MBP–Vav1 C1–KR cannot interact with other lipids that do not belong to the PI family (Figure 6A, second panel from left). We also observed that the MBP–C1–KR–like (KRL) region of Vav3 (Figure 5A and Figure S4G) displays very low affinity towards PI3P and cannot interact with the rest of the lipids tested (Figure 6A, third panel from left). This indicates that the spatial arrangement of the basic amino acids rather than the overall pI of the KR region determines the high affinity towards mono-PIs. The inclusion of the nonchimeric MBP in these experiments (Figure S4G), which cannot bind any of the lipids surveyed (Figure 6A, left panel), confirmed that the binding observed with the fusion proteins is Vav1-specific. These results indicate that the entire Vav1 C1–KR cassette is required for optimal phospholipid binding (Figure 6B).

### 3.7. PI5P Contributes to Vav1 Activity in a KR Region-Dependent Manner in Lymphocytes

**Figure 7:**
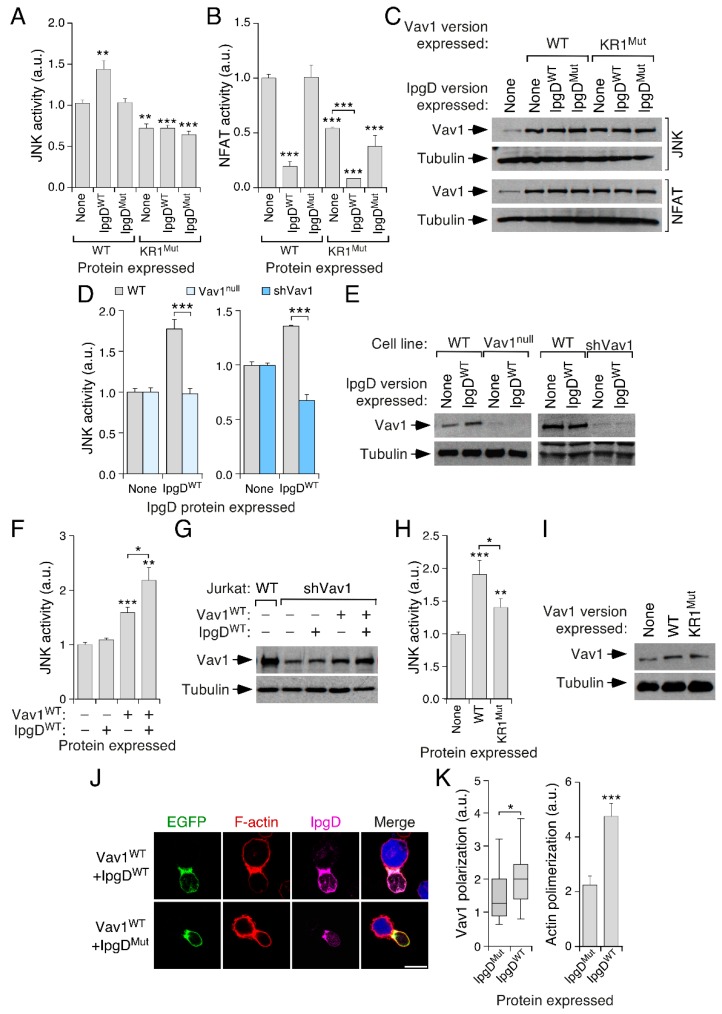
Phosphatidylinositol 5-phosphate (PI5P) contributes to Vav1 activity in Jurkat cells in a KR region-dependent manner. (**A**,**B**) Activation of JNK (**A**) and NFAT (**B**) by the indicated combination of Vav1 and IpgD proteins in nonstimulated Jurkat cells. Data represent, in both cases, the mean ± SEM. Statistical values obtained using the Mann–Whitney U test are given relative to the data obtained in Vav1^WT^-expressing cells. *n* = 3 (**A**) and 2 (**B**) independent experiments, each performed in triplicate. (**C**) Representative immunoblots showing the abundance of the ectopic proteins and endogenous tubulin α in the experiments shown in A (two top blots) and B (two bottom blots), respectively. (**D**) Stimulation of JNK by the expression of IpgD in WT, *VAV1* knockout (*VAV1*^−/−^, left panel), and *VAV1* knockdown (sh*VAV1*, right panel) Jurkat cells. Data represent, in all cases, the mean ± SEM. Statistical values calculated using the Mann–Whitney U test are given relative to the data obtained in IpgD-expressing WT Jurkat cells. *n* = 4 (left panel) and 1 (right panel) independent experiments, each performed in triplicate. (**E**) Representative immunoblots showing the abundance of the endogenous Vav1 and tubulin α in the experiments shown in D. (**F**,**H**) Activation of JNK in *VAV1* knockdown Jurkat cells expressing the indicated combinations of Vav1 (F,H) and IpgD (**F**) proteins. Data represent, in both cases, the mean ± SEM. Statistical values obtained using the Mann–Whitney U test are given relative to the data obtained in Vav1^WT^-expressing cells (*n* = 3). (**G**,**I**) Representative immunoblots showing the abundance of the endogenous Vav1 and tubulin α in the experiments shown in F (**G**) and H (**I**). (**J**,**K**) Example (**J**) and quantitation (**K**) of the localization of ectopically expressed EGFP-tagged Vav1^WT^ (**K**, **left**) and the polymerization of F-actin (**K**, **right**) in the immune synapse formed by Jurkat and Raji cells in the presence of the indicated versions of IpgD (*n* = 3 independent experiments).

Finally, we addressed the role of the PI monophosphates in Vav1 signaling. We focused on PI5P, as previous studies have reported both its upregulation in the plasma membrane of stimulated T cells and its implication on T cell signaling [6,7,8]. To this end, we evaluated the impact on Vav1 activity of the coexpression of IpgD, a *Shigella* PI(4,5)P_2_ phosphatase that promotes PI5P production from PI(4,5)P_2_ when introduced in eukaryotic cells [53,54,55]. The expression of this phosphatase promotes an elevation of Vav1^WT^ activity when tested in JNK assays in Jurkat cells. By contrast, it does not have any effect on the biological activity of Vav1 KR1^Mut^ (Figure 7A). The expression of a catalytically inactive version of IpgD eliminates the positive effect on Vav1^WT^ activity seen with IpgD^WT^ (Figure 7A), further indicating that this increase in Vav1 signaling is PI5P-dependent. We could not measure the effect of IpgD on Vav1-mediated NFAT activity, because its ectopic expression leads to the inhibition of NFAT signaling in a catalysis-dependent manner (Figure 7B). This is probably because of the IpgD catalysis-mediated reduction in the pool of intracellular PI(4,5)P_2_, the substrate used by PLCγ1 to generate the second messengers required for the stimulation of the NFAT pathway (Figure 1B). The abundance of the ectopic Vav1 proteins used in these experiments was confirmed using immunoblot analyses (Figure 7C).

To further corroborate those data, we measured the ability of the ectopically expressed IpgD phosphatase to stimulate JNK in the presence and absence of endogenous Vav1. As shown in Figure 7D,E, this phosphatase can trigger the stimulation of JNK activity in WT, but not in Vav1^null^ or in *VAV1* knockdown (shVav1) Jurkat cells. Such activity, however, is rescued when Vav1^WT^ is re-expressed in the *VAV1* knockdown cells (Figure 7F,G). According to the previous data shown in this work, we also found that Vav1^WT^ elicits higher levels of activation than the Vav1 KR1^Mut^ when ectopically expressed in the *VAV1* knockdown Jurkat cells (Figure 7H,I). Finally, we observed that the expression of IpgD^WT^, but not of the catalytically inactive mutant counterpart, increased both the localization of Vav1^WT^ and the polymerization of F-actin at the immune synapse (Figure 7J,K). Taken together, our results indicate that the entire C1–KR cassette contributes to the optimal biological activity of Vav1 in a PI5P-dependent manner. Finally, we observed using specific bioreporters that PI5P and PI4P, but not PI3P, could localize in the immune synapse. Such localization is not disturbed when tested in *VAV1* knockdown Jurkat cells (Figure S5). This suggests that the expression of Vav1 is not required to maintain the steady-state levels and normal subcellular distribution of those three mono-PIs in Jurkat cells.

## 4. Discussion

**Figure 8:**
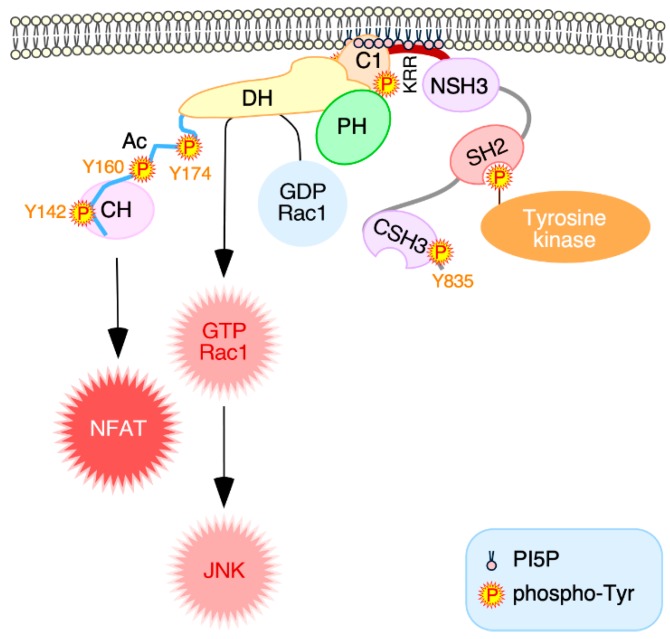
A model for the PI5P-mediated regulation of Vav1 downstream signaling. Upon the SH2-mediated activation of Vav1 by upstream tyrosine kinases, the active Vav1 molecules become stably associated with areas of the plasma membrane enriched in PI5P and, possibly, other mono-PIs. This favors both the optimal signaling output and the diversification activities of active Vav1 proteins, as the mono-PI-mediated regulation impacts more significantly in the levels of stimulation of the NFAT pathway. Specific icons used in the figure are explained in the blue inset. Other abbreviations have been described in the main text.

Here, we have reported a new, PI5P-mediated regulatory layer that contributes to the specific signaling output of Vav1 in lymphocytes (Figure 8). This regulatory step, which is not conserved in the family paralogs, is hierarchically located downstream of the canonical SH2- and phosphorylation-dependent mechanism of activation of Vav1. Consistent with this, our data indicate that it is connected to the regulation of the stability of active Vav1 at both the plasma membrane and the immune synapse (Figure 8). This regulatory layer specifically impacts the signaling diversification properties of this protein, as it has a more pronounced impact on the Vav1 signaling branch that leads to the activation of NFAT (Figure 8). This suggests that, although the NFAT and the Rac1–JNK pathways become concurrently stimulated upon the Vav1 tyrosine phosphorylation step [16], the full engagement of the former route must require longer periods of localization of the protein at the plasma membrane than the JNK-associated pathway. The basis of this signaling selectivity is unknown. It could be related to the fact that the NFAT pathway needs the stepwise engagement of a much larger number of signaling elements than the Rac1–JNK axis to achieve full activation [16,22]. Alternatively, it is possible that the interaction with PI5P molecules could favor the acquisition of a specific orientation of Vav1 at the plasma membrane that could facilitate the interaction with the proximal effectors that are involved in the stimulation of the NFAT pathway. In this context, we have shown before that the engagement of the NFAT response requires specific structural arrangements of Vav1 that are not needed for the efficient triggering of its catalysis-dependent pathways [42].

The binding of PI5P and other mono-PIs to Vav1 requires a bipartite motif composed of the atypical Vav1 C1 domain and an adjacent polybasic tail. Each of those regions shows per se very weak activity and limited specificity towards PI3P. However, when combined, the resulting cassette exhibits much higher binding affinities and, in addition, a broader specificity towards PI5P and, to a lesser extent, PI4P. These features suggest that these two regions form a single, mono-PI binding structure. The requirement of these two motifs also explains the Vav family specificity of this regulatory step, as all the known Vav family paralogs lack a polybasic region downstream of the C1 domain. These results, together with the coevolution of the Vav1 KR region with the adaptive immune system discussed above, further suggest that this new regulatory layer has been developed ad hoc to accommodate the stringent conditions required for the stimulation of Vav1 in lymphocytes. The combined requirement of a zinc finger domain and a polybasic tail is, to our knowledge, a structural feature of Vav1 that is not shared by other mono-PI-binding proteins. However, it bears some resemblance with the case of PHD-containing proteins. Indeed, many proteins containing this alternative zinc finger module also exhibit a polybasic tail (Figure S6). The effective binding of mono-PIs to some of these PHD-containing proteins (e.g., Ing2, Taf3, Sap30 family proteins) also requires the integrity of the PHD-polybasic tail cassette as in the case of Vav1 [33,56,57]. These results indicate that these two mono-PI binding motifs represent independent evolutionary and structural solutions to fulfill the same regulatory need. However, it is worth noting that such mechanisms are not universal, because other PHD proteins only require the polybasic tail for effective lipid binding [58]. Our results also raise the question of whether other C1 domains, especially those belonging to the atypical subtype, might perform the same regulatory function in other signaling proteins. The analysis of all the C1 domain-containing proteins encoded by the human genome indicates that the large majority of them lack polybasic tails (Table S2). The only exceptions include Vav1 and two members of the diacylglicerol kinase family (DGK ι and ζ) (Table S2). It will be interesting to test whether these proteins could be also subjected to a Vav1-like regulatory mechanism.

## Supplementary Materials

The following are available online at https://www.mdpi.com/2073-4409/8/12/1649/s1.
