# Supplementary material for: Phosphatidylinositol Monophosphates Regulate Optimal Vav1 Signaling Output
Source: Cells. 2019 Dec 16;8(12):1649. doi: 10.3390/cells8121649 (PMC6952945; doi:10.3390/cells8121649)
Supplement: Supplementary file 1 [file cells-08-01649-s001.pdf]

Supplemental Information for

PHOSPHATIDYLINOSITOL MONOPHOSPHATES REGULATE OPTIMAL Vav1 SIGNALING OUTPUT

by

Sonia Rodríguez–Fdez, Carmen Citterio, L. Francisco Lorenzo–Martín, Jesús Baltanás–Copado, Clara Llorente–González, Senena Corbalán–García, Miguel Vicente–Manzanares, and Xosé R. Bustelo*

*To whom correspondence should be addressed. E–mail: xbustelo@usal.es

This PDF file includes:

(1) Supplemental Figures S1 to S6 and legends (pages 2 to 10)

(2) Supplemental Tables S1 and S2 (pages 11 to 13)

	Uniprot code	Species	Amino acid sequence	Charge +	Charge −	Charge Net	pI
Cnidaria	T2MGD6	*Hydra vulgaris* Vav	------------KPPSKPITSKPTIHLRT----				
Nematoda	Q45FX5	*Caenorhabditis elegans* Vav	ET----------RSSQSFNCNRP---RFHIH--				
Hexapoda	Q9NHV9	*Drosophila melanogaster* Vav	--KQPNPVSVPPPVHDTQYALSL--KTDNTVK				
Chondrichthyes	V9KF60	*Callorhinchus milii* Vav1	NTFKKSK-SS-RAPPPRQPNTDLP-KMEVLQE	6	2	+4	10.45
Teleostei	H2M046	*Oryzias latipes* Vav1	GTSRIIKVSL-HLSSKLKYFYGFP-KMEVSQE	6	1	+5	10.00
	I3IUK0	*Oreochromis niloticus* Vav1	VSAKKNK----AQRSSGHSSIGYP-KMEVNQE	6	1	+5	10.17
	H2UD65	*Takifugu rubripes* Vav1	GTIKKVKTHINIFSPPLRSMLGFP-KMEVCQD	6	1	+5	10.46
Amphibia	Q6DCX0	*Xenopus laevi* Vav1	STNKKQK-TG-RGNNSKGNEKGLP-KMEACEE	7	2	+5	10.30
Reptilia	A0A1U7RH95	*Alligator sinensis* Vav1	SVLKKNKSNR-HTQEKRKNDLGLP-KMEVCQD	9	3	+6	10.29
Mammalia	P27870	*Mus musculus* Vav1	GTMKKDKLHR-RAQDKKRNELGLP-KMEVFQE	10	4	+6	10.28
	Q08DN7	*Bos taurus* Vav1	GTMKKDKPHR-RAQDKKRNELGLP-KMEVCQE	10	4	+6	10.28
	P15498	*Homo sapiens* Vav1	GTMKKDKLHR-RAQDKKRNELGLP-KMEVFQE	10	4	+6	10.28
	P52735	*Homo sapiens* Vav2	---------------DASGAGPG-P-KMVAMQN				
	Q9UKW4	*Homo sapiens* Vav3	GTLKLPEKRTNGLRRTPKQVDPGLPKMQVIR-	7	2	+5	11.07

FIGURE S1. Evolution of the KR in Vav family proteins

Amino acid sequence and charge features of the Vav1 KR region were analyzed in the indicated species. Positively charged residues are shown in red.

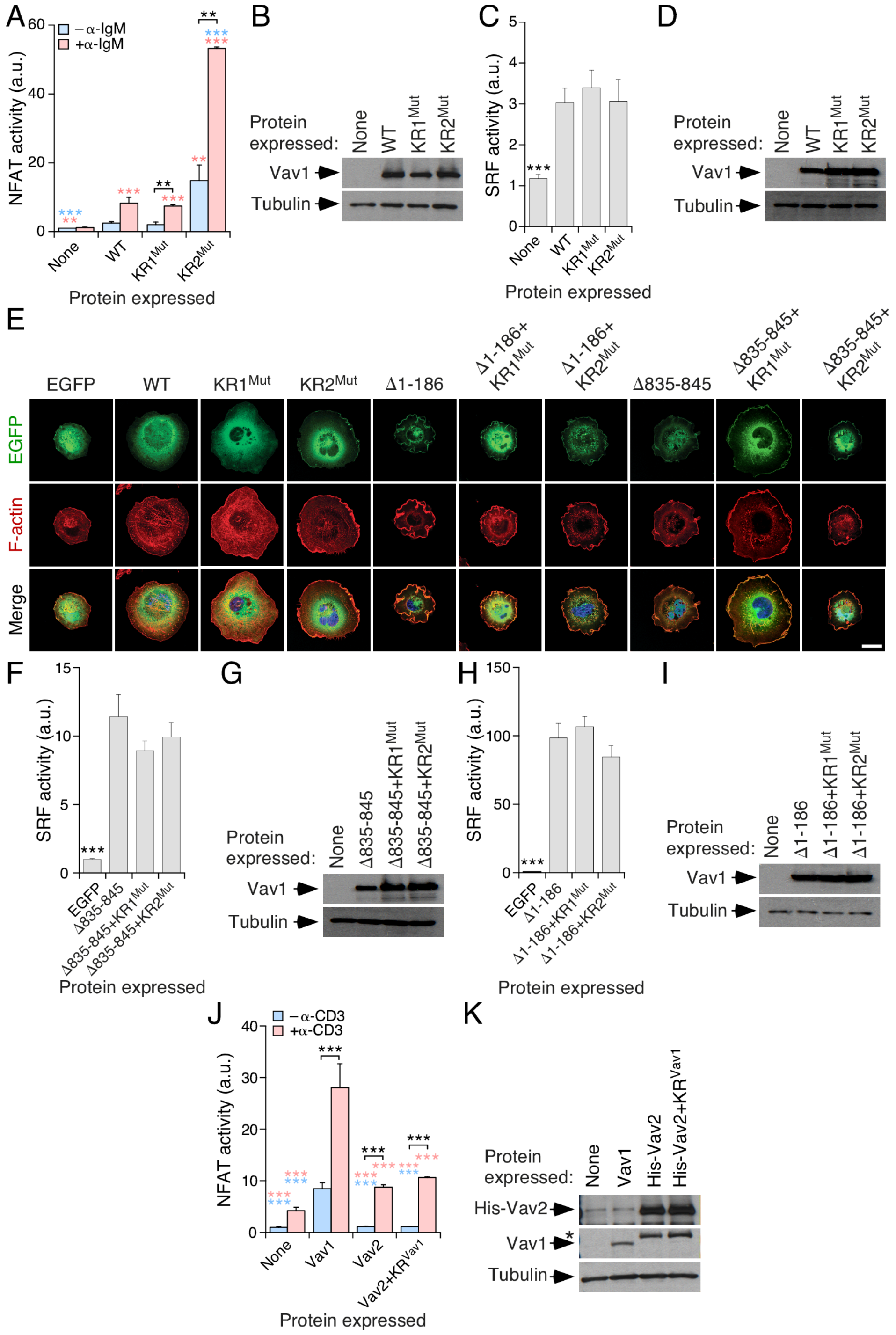

FIGURE S2. The Vav1 KR regulatory mechanism is specific for lymphocytes

(A) Activation of NFAT triggered by indicated Vav1 proteins in nonstimulated and BCR–stimulated DT40 cells. Data represent the mean ± SEM. Statistical values were obtained using the Mann–Whitney U test. Blue and salmon asterisks indicate the significance level compared with nonstimulated and BCR–stimulated Vav1^{WT}–expressing cells, respectively. Black asterisks refer to the *P* values obtained between the indicated experimental pairs (in brackets). *n* = 3 independent experiments.

(B) Representative example of the abundance of the indicated Vav1 proteins and tubulin α (loading control) in the assays performed in A.

(C) Activation of SRF by the indicated Vav1 constructs in COS1 cells. Data represent the mean ± SEM. Statistical values were obtained using the Mann–Whitney U test. All the comparisons are referred to $Vav1^{WT}$. *n* = 3 independent experiments, each performed in triplicate.

(D) Representative example of the abundance of the indicated Vav1 proteins and tubulin α (loading control) in the assays performed in C.

(E) Effect of the indicated proteins (top) in the F–actin cytoskeleton of COS1 cells. EGFPs and F–actin are shown in green and red, respectively. Areas of colocalization of Vav1 proteins and F–actin are shown in yellow (bottom panels). Scale bar, 20 μm. *n* = 3 independent experiments.

(F and H) Activation of SRF by the indicated Vav1 proteins in COS1 cells. Data represent the mean ± SEM. Statistical values were generated applying the Mann–Whitney U test using as comparative control the values obtained in $Vav1^{\Delta 835-845}$– (F) and $Vav1^{\Delta 1-186}$–expressing (H) cells. *n* =3 independent experiments, each performed in triplicate.

(G and I) Representative example of the abundance of the indicated Vav1 proteins and tubulin α (loading control) in the assays performed in F (G) and H (I).

(J) Activation of NFAT triggered by $Vav1^{WT}$ and indicated polyhistidine–tagged Vav2 (His–Vav2) proteins in nonstimulated and TCR–stimulated Jurkat cells. Data represent the mean ± SEM. Statistical values were obtained using the Mann–Whitney U test. Blue and salmon asterisks indicate the significance level compared with nonstimulated and TCR–stimulated $Vav1^{WT}$–expressing cells, respectively. Black asterisks refer to the *P* values obtained between the indicated experimental pairs (in brackets). *n* = 3 independent experiments, each performed in duplicate.

(K) Representative example of the abundance of the indicated Vav proteins and endogenous tubulin α (loading control) in the assays performed in J. Vav2 was detected using an antibody to the polyhistidine tag. The asterisk pinpoints the residual signal from the previous blotting carried out with the antibody to polyhistidine residues.

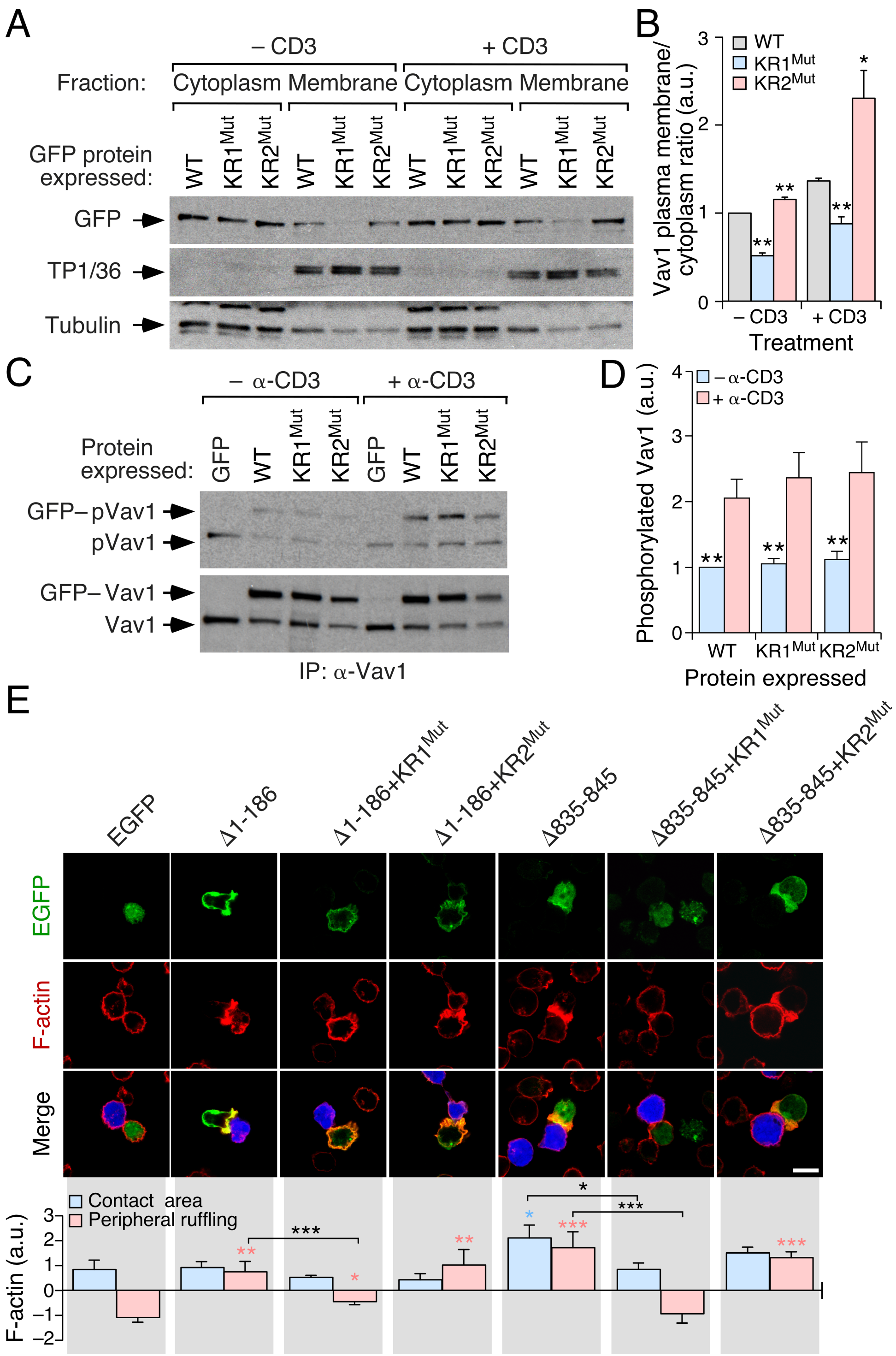

FIGURE S3. The KR region is important for the localization of Vav1 in lymphocytes

(A) Immunoblot of cytoplasmic and plasma membrane fractions of nonstimulated and CD3–stimulated Jurkat cells showing the localization of the indicated EGFP–tagged Vav1 versions (top), a membrane–localized (TP1/36), and a cytosolic (tubulin α) protein. Please, note that the tubulin panel was generated using aliquots from the same experiment in an independent filter.

(B) Quantification of the distribution of the indicated Vav1 proteins in the membrane and cytosolic fractions of nonstimulated and stimulated Jurkat cells from the experiments shown in A. Data are shown as mean ± SEM. Statistical values were calculated using the Student's *t* test relative to the data obtained in $Vav1^{WT}$–expressing cells. $n = 3$ independent experiments.

(C) Example of the tyrosine phosphorylation of the indicated ectopically-expressed (GFP-pVav1) and endogenous Vav1 (pVav1) proteins in nonstimulated and TCR-stimulated Jurkat cells (top panel). As control, we include the Western blot of immunoprecipitated Vav1 with antibodies to the Vav1 DH domain (bottom panel).

(D) Quantification of the tyrosine phosphorylation levels of the indicated ectopically-expressed Vav1 proteins obtained in the experiments shown in C. Data are shown as mean ± SEM. Statistical values were calculated using the Student's *t* test relative to the data obtained in $Vav1^{WT}$–expressing cells. $n = 6$ independent experiments.

(E) Example (top panels) and quantification (bottom) of the effect of EGFP and indicated EGFP–tagged Vav1 versions (top) in the polymerization of actin inside the contact area and outside or at the peripheral area of the immune synapse. This data is part of the same experiment performed in **Figure 3G**. As in that case, values were obtained comparing the F–actin signal in these areas to the signal in other regions of both the T and B cell as detailed in the methods. Histograms represent the mean ± SEM and statistics were performed using two–way ANOVA and Dunnett's multiple comparison tests using as reference control the detection of F–actin inside (blue) and outside (salmon) the contact area in Jurkat cells expressing the indicated EGFP–protein. $n = 3$ independent experiments.

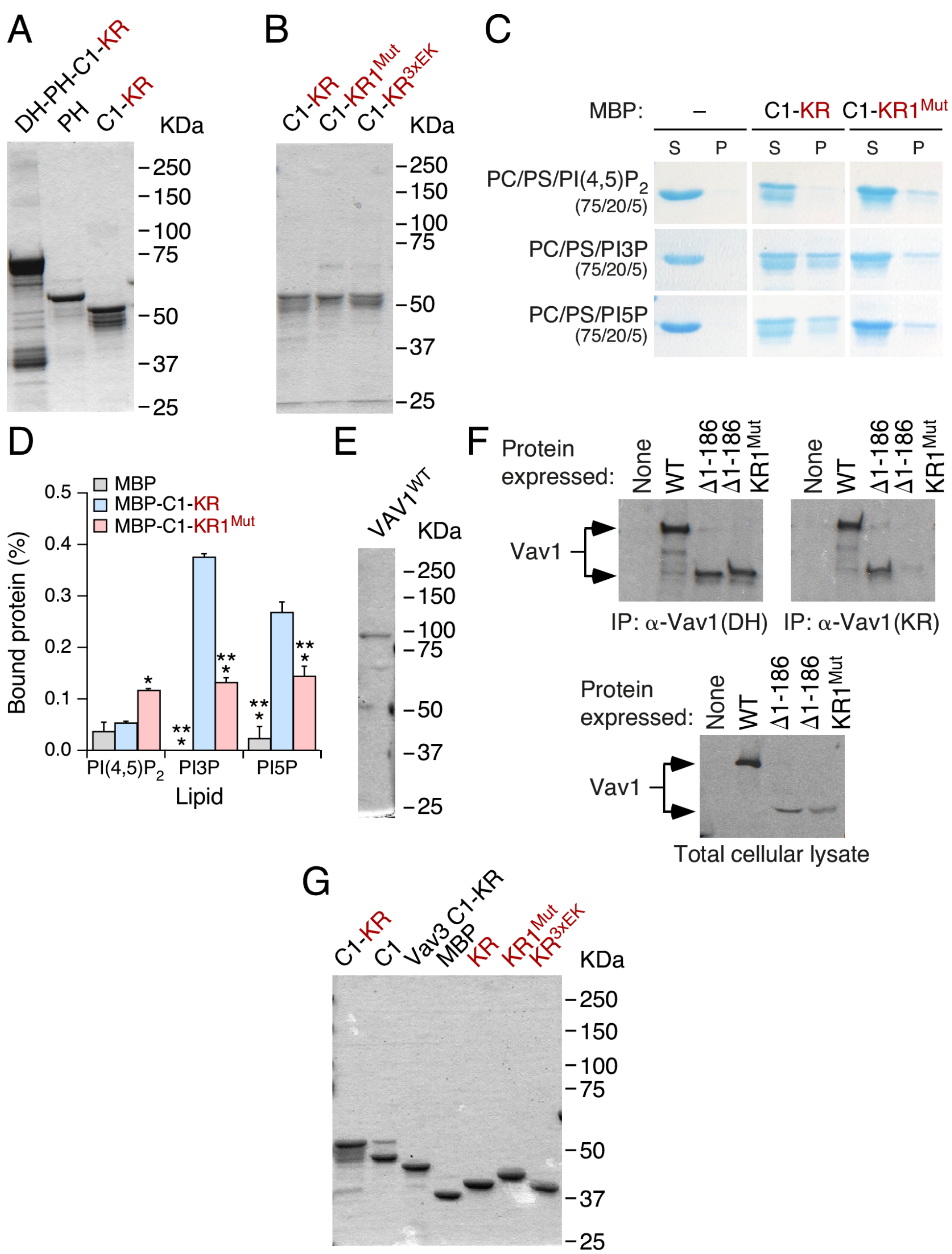

FIGURE S4. The entire Vav1 C1–KR mediates phosphatidylinositol monophosphate binding

(A,B) Stained SDS–PAGE gels showing the purified MBPs used in **Figure 5B,C**. The migration of the molecular weight markers is shown on the right of each panel. KDa, kilodalton.

(C) Representative experiment showing the association of the specified MBP proteins (top) with the liposomes of the indicated composition (left). S, soluble (unbound) fraction; P, pelleted (bound) fraction. PC, 1–palmitoyl–2–oleoyl–sn–glycero–3–phosphocholine; PS, 1–palmitoyl–2–oleoyl–sn–glycero–3–phospho–L–serine. Proteins were stained with Coomassie.

(D) Quantification of the experiments shown in C. Data represent the mean ± SEM. Statistical values were obtained using two–way ANOVA followed by Dunnett's test for multiple comparisons. n = 5 independent experiments.

(E) Stained SDS–PAGE gel showing the purified Vav1^{WT} protein used in **Figure 5E**. The migration of the molecular weight markers is shown on the right.

(F) Immunoprecipitation of indicated Vav1 proteins (top) with antibodies to epitopes located in the Vav1 DH domain (top panel on the left) and KR (top panel on the right). As control, we show the expression of each of the protein versions used aliquots of the total cellular lysates used in the immunoprecipitation experiments (bottom panel).

(G) Stained SDS–PAGE gels showing the purified MBPs used in **Figure 6**. The migration of the molecular weight markers is shown on the right of each panel.

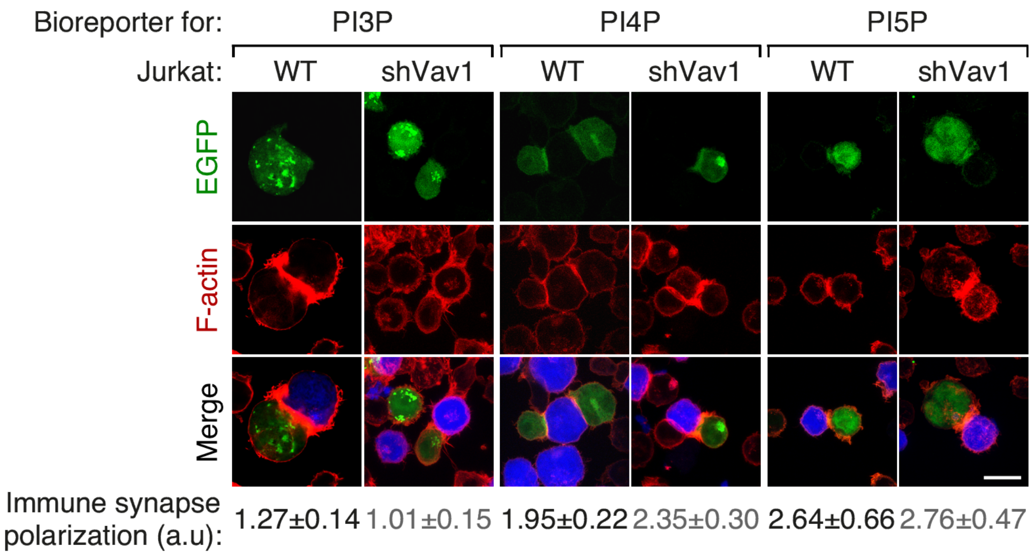

FIGURE S5. Vav1 does not influence the localization of phosphatidylinositol monophosphates at the immune synapse.

WT and VAV1 knockdown (shVav1) Jurkat cells were transfected with EGFP-tagged domains that specifically recognize PI3P (NAPD oxidase PX domain), PI4P (four phosphate adaptor protein 1 PH domain) and PI5P (Ing2 PHD domain). Upon synapse formation, cells were fixed, stained with phalloidin, and subjected to confocal microscopy. The bioreporters and F-actin are seen as green and red signals. Areas of colocalization between the bioreporters and F-actin are seen in yellow.

Uniprot code	Protein	Amino acid sequence
P15498	Vav1 (529-592)	CKACQMLLRGTFYQGYRCHR--CR-ASAHKECLG---------RVPPCGRHGQDFPGTMKKDKLHRRAQDKKRNEL
Q5VWG9	Taf3 (868-929)	CPGCNKPDDGS--PMIGCDD--CD-DWYHWPCVG-IMTAPPEEMQWFCPKCAN-----KKKDKKHKKRKHRAH---
Q96T88	Uhrf1 (318-388)	CHLCGGRQDPD--KQLMCDE--CDMAFHIY-CLDPPLSSVPSEDEWYCPECRNDASEVVLAGERLRESKKKAKMAS
Q9H160	Ing2 (215-280)	CL-CNQVSYG---EMIGCDNEQCPIEWFHFSCVS--LTYKPKG-KWYCPKCRGDNEKTMDKSTEKTKKDRRSR---
Q9HAJ7	Sap30L (29-99)	C--CLIEDGERCVRPAGNASFSKRVQKSISQKKLKLDIDKSVRHLYICDFHKN---FIQSVRNKRKRKTSDDGGDS
		ZF regions / Polybasic regions

FIGURE S6. Comparison of the Vav1 C1–KR with the PHD–KR cassette of nuclear proteins

The residues involved in the coordination of Zn^{2+} are shown in green and shaded in dark gray. Basic residues present in the KR regions of the indicated proteins are shown in red. The basic residues present in the Ing2 PHD that contribute to binding to phosphatidylinositol monophosphates are also indicated in red.

TABLE S1. Sequence of oligonucleotides used in this study

Mutant		DNA sequence of primer
KR1[Mut]	F_1*	5'– CAAGATTTCGCAGGAACCAT(G)GAAGAAGGACAAGCTCCATC –3'
	R_1	5'– GATGGAGCTTGTCCTTCTTC(C)ATGGTTCCTGCGAAATCTTG –3'
	F_2	5'– GGACAAGCTCCATCG–AGGGCCCAGGACAAG –3'
	R_2	5'– CTTGTCCTGGGCCCT–CGATGGAGCTTGTCC –3'
KR2[Mut]	F_1	5'– CATCGAAGGGCCCAGGAC–AGAAAAGGAATGAATTGGG –3'
	R_1	5'– CCCAATTCATTCCTTTTCT–GTCCTGGGCCCTTCGATG –3'
	F_2	5'– TCTGCCTAAGATGGAAGTGTTT(T)CAGGAATACTATGGGATCCCTC –3'
	R_2	5'– GAGGGATCCCATAGTATTCCTG(A)AAACACTTCCATCTTAGGCAGA –3'
CAAX	F_1	5'– GCCTGGTGCCCTGTAGGACACAGCGAG –3'
	R_1	5'– [p]–GCTCTAGATCAGGAGAGGACGCAGCAATATTCGGAATAGTCTTCC –3'
	F_2	5'– GGAAGACTATTCCGAATATTGCTGTATGTCATGTAAGTGCGTCCTCTCCTG ATCTAGAGC –3'
	R_2	5'– GCTCTAGATCAGGAGAGGACGCACTTACATGACATACAGCAATATTCGGA ATAGTCTTCC –3'
Δ835–845	F	5'– CCCTTCTAACTAAGTGGAGGAAGAC –3'
	R	5'– GTCTTCCTCCACTTAGTTAGAAGGG –3'
D578K	F	5'– CGCAGGAACCATGAAGAAGAAAAAGCTCCATCGAAGGGCCC –3'
	R	5'– GGGCCCTTCGATGGAGCTTTTTCTTCTTCATGGTTCCTGCG –3'
E591K	F	5'– CCCAGGACAAGAAAAGGAATAAATTGGGTCTGCCTAAGATG –3'
	R	5'– CATCTTAGGCAGACCCAATTTATTCCTTTTCTTGTCCTGGG –3'
E598K	F	5'– GGGTCTGCCTAAGATGAAAGTGTTTCAGGAATAC –3'
	R	5'– GTATTCCTGAAACACTTTCATCTTAGGCAGACCC –3'
G691V	F	5'– CCAACCGTTCTGATGTGACCTATCTGGTGCG –3'
	R	5'– CGCACCAGATAGGTCACATCAGAACGGTTGG –3'
MBP–C1	F	5'– GCAGGAACCATGTAGAAGGACAAGC –3'
	R	5'– GCTTGTCCTTCTACATGGTTCCTGC –3'
MBP–KR	F	5'– CCTATAGATCTGGTCGCCATGGGCAAGATTTCGC –3'
	R	5'– GGAGGAGGGATCCCATAGTATTCC –3'

*F, forward primer (in the case of a two–step mutagenesis protocol, the primers are referred to as F_1 and F_2 for the first and second step, respectively); R, reverse primer (in the case of a two–step mutagenesis protocol, the primers are referred to as R_1 and R_2 for the first and second step, respectively); [p]–, phosphorylated primer. Nucleotides used for the generation of the indicated mutations are shown in red. Nucleotides that have been inserted or deleted in the WT sequence are indicated in red parenthesis and with a red line, respectively.

TABLE S2. Alignment of the amino acid sequences present at the C–terminus of C1 domains (continues in next page)

Protein	Region C–terminal to C1 domain*
Vav1	GRHGQDFPGTMKKDKLHRRAQDKKRNELGLPKMEVFQEYYGLPPPPGAIG
DGKι	KPTFREGGSRSPRENFVRHHWVHRRRQEGKCKQCGKGFQQKFSFHSKEIV
DGKς	KPSFRESGSRNVREPTFVRHHWVHRRRQDGKCRHCGKGFQQKFTFHSKEI
DGKθ	SDCRQCHQDGHQDHDTHHHHWREGNLPSGARCEVCRKTCGSSDVLAGVRC
RasGRP2	RRRAQSVSLEGSAPSPSPMHSHHHRAFSFSLPRPGRRGS
PKCα	PGADKGPDTDDPRSKHKFKIHTYGSPTFCDHCGSLLYGLIHQGMKCDTCD
PKCα (isoform 2)	GSSGGPDTDDPRSKHKFKIHTYGSPTFCDHCGSLLYGLIHQGMKCDTCDM
PKCβ	PGADKGPASDDPRSKHKFKIHTYSSPTFCDHCGSLLYGLIHQGMKCDTCM
PKCγ	PGAGKGPQTDDPRNKHKFRLHSYSSPTFCDHCGSLLYGLVHQGMKCSCCE
DGKγ	CVKTYSKAKRSGEFHRKCELSTLCDGGELRDHILLPTSICPITCVKTYSK
DGKβ	CIKTYVKSKRNTDVMHHYWVEGNCPTKCDKCHKTVKCYQGLTGLHCVWCQ
DGKα	EVSTYAKSRKDIGVQSHVWVRGGCESGRCDRCQKKIRIYHSLTGLHCVWC
Vav3	GRVNSGEQGTLKLPEKRTNGLRRTPKQVDPGLPKMQVIRNYSGTPPPALH
RasGRP3	RRFARAPSLSSGHGSLPGSPSLPPAQDEVFEFPGVTAGHRDLDSRAITLV
RasGRP1 (isoform 2)	KKRAKNPVAPTENNTSVGPVSNLCSLGAKDLLHAPEEGPFTFPNGEAVEH
RasGRP4	KKRPGAKGDAGPPGAPVPSTPAPHASCGSEENHSYTLSLEPETGCQLRHA
PKD3	SGVRKRRLSNVSLPGPGLSVPRPLQPEYVALPSEESHVHQEPSKRIPSWS
PKD1	SGVRRRRLSNVSLTGVSTIRTSSAELSTSAPDEPLLSPVSPGFEQKSPSE
PKD2 (fragment)	DCKFNCHKRCATRVPNDCLGEALINGDVPMEEATDFSEADKSALMDESED
PKCς	RKHMDSVMPSQEPPVDDKNEDADLPSEETDGIAYISSSRKHDSIKDDSED
DGKδ	KWTTLASIGKDIIEDADGIAMPHQWLEGNLPVSAKCTVCDKTCGSVLRLQ
DGKδ (isoform 2)	TTLASIGKDIIEDADGIAMPHQWLEGNLPVSAKCTVCDKTCGSVLRLQDW
DGKη	KWTTLASIGKDIIEDEDGVAMPHQWLEGNLPVSAKCAVCDKTCGSVLRLQ
DGKκ	KWNTLSITDDLLLPADEVNMPHQWVEGNMPVSSQCAVCHESCGSYQRLQD
DGKε	KEIMLKNDTKVLDAMPHHWIRGNVPLCSYCMVCKQQCGCQPKLCDYRCIW
DEF8	VSSKVSHQAEYELNICPETGLDSQDYRCAECRAPISLRGVPSEARQCDYT
PLEKHM3	SKYKVSKQAKEFLEYVYEEPLIDIQQENAMLYHHAEPLAAVLRLRQRLKS
B–RAF	VNYDQLDLLFVSKFFEHHPIPQEEASLAETALTSGSSPSAPASDSIGPQI
TENC1	QALPPVELRRNTAPVRRIEHLGSTKSLNHSKQRSTLPRSFSLDPLMERRW
PNS3	GVQVRLEQAPGSSTLSSSLCRDKPLRPVILSPTMEEGHGLDLTYITERII
RhoGEF28	TKKFQEKYNKNKPQTILGNSSFRDIPQPGLSLHPSSSVPVGLPTGRRETV
ADCP5	SQQEGLSRDRPSPESTLTVTFSQNVCKPVEETQRPPTLQEIKQKIDSYNT
RacGAP1	IPTLIGTPVKIGEGMLADFVSQTSPMIPSIVVHCVNEIEQRGLTETGLYR
AKAP13	AKVKMKQPKGSLQAHDTSSLPTVIMRNKPSQPKERPRSAVLLVDETATTP
RAF	VDWSNIRQLLLFPNSTIGDSGVPALPSLTMRRMRESVSRMPVSSQHRYST
RAF (isoform X5)	VDWSNIRQLFSQHRYSTPHAFTFNTSSPSSEGSLSQRQRSTSTPNVHMVS
PKCι	GRHSLPQEPVMPMDQSSMHSDHAQTVIPYNPSSHESLDQVGEEKEAMNTR
KMT2C	GRQRLPFSAPPGSVVEASSNLRHGNFIPRPDFPGPRHTDPMRRPPQGLPN
ARAF	VDMSTNRQQPSRFYHSVQDLSGGSRQHEAPSNRPLNELLTPQGPSPRTQH
TNS1	VPPSNHELVPITTENAPKNVVDKGEGASRGGNTRKSLEDNGSTRVTPSVQ
ADCP1	CGPRDLGWEPAVERDTNVDEPVEWETPDLSQAEIEQKIKEYNAQINSNLF
PDZD8	GATDRRIDRTLKNLRLEGQETLLGLPPRVDAEASKSVNKTTGLTRHIINT
Vav2	KFTSPADLDASGAGPGPKMVAMQNYHGNPAPPGKPVLTFQTGDVLELLRG

*Basic amino acid residues are shaded in red.

TABLE S2 (continuation from previous page)

Protein	Region C–terminal to C1 domain
UNC13B	LQRAAEKSCKHGAEDRTQNIIMAMKDRMKIRERNKPEIFEVIRDVFTVNK
UNC13A	LQRAAEKSSKHGAEDRTQNIIMVLKDRMKIRERNKPEIFELIQEIFAVTK
UNC13C	LQRAAEKSSKHGAEDKTQTIITAMKERMKIREKNRPEVFEVIQEMFQISK
STAC	MGKLPKGFRRYYSSPLLIHEQFGCIKEVMPIACGNKVDPVYETLRFGTSL
STAC2	PGKTSTSFRRNFSSPLLVHEPPPVCATSKESPPTGDSGKVDPVYETLRYG
STAC3	FGKIPPGFHRAYSSPLYSNQQYACVKDLSAANRNDPVFETLRTGVIMANK
KSR2	LLIIHRGDPARLVRTESVPCDINNPLRKPPRYSDLHISQTLPKTNKINKD
KSR1	ISFLPLTRLRRTESVPSDINNPVDRAAEPHFGTLPKALTKKEHPPAMNHL
PKCδ	TGTAANSRDTIFQKERFNIDMPHRFKVHNYMSPTFCDHCGSLLWGLVKQG
PKCζ	TGSAINSRETMFHKERFKIDMPHRFKVYNYKSPTFCEHCGTLLWGLARQG
PKCε	AGLKKQETPDQVGSQRFSVNMPHKFGIHNYKVPTFCDHCGSLLWGLLRQG
PKCη	TCQNNINKVDSKIAEQRFGINIPHKFSIHNYKVPTFCDHCGSLLWGIMRQ
PKCθ	GKIDMPHRFKVYNYKSPTFCEHCGTLLWGLARQGLKCDACGMNVHHRCQT
MRCKα	PVPPEQTKGPLGIDPQKGIGTAYEGHVRIPKPAGVKKGWQRALAIVCDFK
MRCKβ (isoform X1)	PIPPEQSKRPLGVDVQRGIGTAYKGHVKVPKPTGVKKGWQRAYAVVCDCK
MRCKγ	PVPPDLLRTALGVHPETGTGTAYEGFLSVPRPSGVRRGWQRVFAALSDSR
ROCK1	SRIEGWLSVPNRGNIKRYGWKKQYVVVSSKKILFYNDEQDKEQSNPSMVL
CIT	GLPAEYATHFTEAFCRDKMNSPGLQTKEPSSSLHLEGWMKVPRNNKRGQQ
PLEKHM1	ESVGPAHSDGRFELVFSGKKLALRASSQDEAEDWLDRVREALQKVRPQQE
CHN1	KPDLKHVKKVYSCDLTTLVKAHTTKRPMVVDMCIREIESRGLNSEGLYRV
CHN2	QPDLKRIKKVYCCDLTTLVKAHNTQRPMVVDICIREIEARGLKSEGLYRV
HMHA1	GHKKLQGRLQLFGQDFSHAARSAPDGVPFIVKKCVCEIERRALRTKGIYR
RhoGAP29	GHQKLPGKIHLFGAEFTQVAKKEPDGIPFILKICASEIENRALCLQGIYR
GMIP	GHRRLPARTPLFGVDFLQLPRDFPEEVPFVVTKCTAEIEHRALDVQGIYR
MYO9A	SKKYDPELSSRQFGVELSRLTSEDRTVPLVVEKLINYIEMHGLYTEGIYR
MYO9B	SYTYGRKGEPGVEPGHFGVCVDSLTSDKASVPIVLEKLLEHVEMHGLYTE
BRD1	LQSRARPA
